# Peripartum Disease in Dairy Cows as a Single LPS-Driven Metabolic-Immune Syndrome: Convergent Metabolomic Evidence Across Six Clinical Presentations

**DOI:** 10.3390/metabo16100743

**Published:** 2026-10-02

**Authors:** Burim N. Ametaj

**Affiliations:** Department of Agricultural, Food and Nutritional Science, University of Alberta, Edmonton, AB T6G 2P5, Canada; burim.ametaj@ualberta.ca

**Keywords:** peripartum disease, transition dairy cow, lipopolysaccharide, mammary involution, metabolic-immune syndrome, metabolomics, kynurenine pathway, TLR4, comorbidity, metaflammation

## Abstract

**Highlights:**

**What are the main findings?**

- Cows later diagnosed with ketosis, lameness, milk fever, metritis, retained placenta, or subclinical mastitis already share thirteen altered serum metabolites eight weeks before calving, including a 2.8–4.0× kynurenine/tryptophan elevation that separates every disease group completely from controls.
- Across the full window from eight weeks before calving to eight weeks after, cross-disease similarity rises to a peak four weeks before calving (r = 0.67), diverges as individual organs fail at diagnosis and +4 wk (r = 0.51 and 0.49), and reconverges by +8 wk (r = 0.64) as cows resolve—a dynamic course, not a fixed cow-level trait.

**What are the implications of the main findings?**

- These six conditions may be alternative clinical exits of one LPS-associated metabolic-immune syndrome, with mammary involution during the dry period as a candidate prepartum source.
- Prevention aimed at the dry-off transition could lower the incidence of all six conditions together, rather than managing each disease separately.

**Abstract:**

Six conditions of the peripartum dairy cow—ketosis, lameness, milk fever, metritis, retained placenta, and subclinical mastitis—are conventionally studied as independent diseases. Here, an integrative re-analysis of metabolomic data from one cohort studied in the author’s laboratory (University of Alberta, Edmonton, Canada), most of them previously published, spanning three biofluids (serum, urine, and milk) and four analytical platforms (DI-LC-MS/MS, GC-MS, ^1^H-NMR, and ICP-MS) evaluates the hypothesis that they are alternative clinical presentations of a single metabolic-immune syndrome whose proposed proximal trigger during the dry-off period is endotoxin (LPS) translocation from the involuting mammary gland, with three concurrent postpartum contributors: continuing mammary involvement and new intramammary infection; gastrointestinal LPS translocation under concentrate-rich feeding; and uterine LPS and Gram-positive lipoteichoic acid absorbed across the involuting endometrium. Four predictions were evaluated. (i) Cross-disease similarity of metabolic effect-size vectors is positive in every matrix, on two complementary serum platforms, and at every timepoint examined (mean Spearman r = 0.35–0.81, lowest postpartum and at clinical diagnosis, where partial divergence is predicted). (ii) Thirteen serum metabolites are altered in the same direction in all six disease groups eight weeks before calving, with the kynurenine/tryptophan ratio elevated 2.8–4.0-fold and completely separating every disease group from controls (*p* = 8.7 × 10^−6^, the minimum attainable for the group sizes). (iii) The urinary metabolome strengthens through the dry-off period (peak concordance at −4 wk, 44 of 91 metabolites) and partially diverges at clinical disease. The disturbance did not appear to resolve with the clinical episode: at 8 weeks postpartum, the mean absolute deviation from contemporaneous controls was similar to that observed 8 weeks before calving (mean |log2 fold change| 0.71 vs. 0.73), and the six diseases still deviated in a shared direction. (iv) Milk shows a postpartum shift consistent with TLR4 signaling (lactate and TCA-cycle intermediates up; betaine down; cobalt up in all six diseases). Comorbidity, conventionally treated as a confounder, may instead be the expected phenotype of one syndrome with multiple vulnerable-organ exits. Because the evidence is correlational and LPS was not measured, the mammary origin remains a hypothesis; direct LPS quantification, source-resolved measurements, and evaluation of competing explanations (glucocorticoid-driven reprogramming, pre-existing nutritional differences, and gut-derived signals) are required. The framework yields falsifiable predictions for dry-off-targeted, syndrome-level prevention.

## 1. Introduction

### 1.1. The Peripartum Disease Complex

The first weeks surrounding parturition are the riskiest period in the life of a dairy cow. During this transition, six conditions account for the majority of disease-related culling and economic loss: ketosis (impaired hepatic adaptation to negative energy balance), lameness (laminitis-related foot pathology), milk fever (parturient hypocalcemia), metritis (uterine inflammation), retained placenta (failure of fetal-membrane release), and subclinical mastitis (intramammary infection without overt clinical signs). Each of these conditions has been the subject of an extensive disease-specific literature, including a substantial body of metabolomic and immunometabolic biomarker work spanning several diseases simultaneously [1] as well as ketosis [2,3,4,5], lameness [6,7,8,9,10,11], milk fever [12,13], metritis [14,15,16,17], retained placenta [18,19,20], and subclinical mastitis [21,22,23,24,25,26]. The implicit assumption underlying virtually all of this literature is that each disease arises from a distinct etiology: a metabolic-energetic failure for ketosis, a calcium-homeostatic failure for milk fever, an infectious-inflammatory failure for metritis and mastitis, a mechanical-inflammatory failure for lameness, a contractile-uterine failure for retained placenta. Prevention and treatment strategies follow this disease-specific logic.

### 1.2. The Puzzle of Comorbidity

A pattern that does not fit the disease-specific paradigm is the high frequency of comorbidity. Cows that develop one peripartum condition are at substantially elevated risk for the others: a cow with milk fever has a several-fold increased risk of retained placenta and metritis [27,28,29]; a cow with ketosis has elevated risk of lameness, displaced abomasum, and metritis [30,31]. The conventional response to this observation is to treat comorbidity as the result of shared general risk factors—negative energy balance, parturition stress, and immunosuppression—that happen to predispose vulnerable cows to several distinct diseases simultaneously [32,33]. This is the framework under which the disease-specific biomarker literature has been built [1,2,3,4,5,6,7,8,9,10,11,12,13,14,15,16,17,18,19,20,21,22,23,24,25,26].

An alternative interpretation, first developed in the context of bacterial endotoxin biology by Eckel and Ametaj [32] and elaborated subsequently [34,35], is restated and tested here: that the high comorbidity rate is not an artifact of overlapping risk factors but the predicted phenotype of a single underlying syndrome that can manifest clinically through whichever organ fails first. Under this hypothesis, the six “diseases” are alternative clinical exits of a common upstream perturbation; comorbidity is expected because the same cow can fail through more than one organ. This framework has recently been extended through a re-interpretation of peripartum hypocalcemia as an adaptive anti-LPS response rather than a primary pathology [36].

### 1.3. Why Mammary Involution Is Proposed as a Leading Candidate LPS Source During the Dry-Off Period

Identifying the upstream trigger requires attention to which physiological challenges actually exist in the cow during the relevant window. The dry-off period—the abrupt cessation of milking approximately 8 weeks before calving—is the most stereotyped physiological challenge that all dairy cows experience and that immediately precedes the disease window. Cessation of milk removal causes intra-alveolar pressure rise, epithelial barrier strain, immune-cell infiltration into the involuting mammary gland [37,38], and a substantial disturbance in the mammary microbial ecology; the involuting gland harbors a dynamic community that can include both Gram-negative and Gram-positive taxa and is particularly susceptible to new intramammary infection and barrier disruption during the early dry period [32,34,39]. The vena lactea drains directly into systemic circulation. The cumulative effect of these processes is hypothesized to be a low-grade, sustained translocation of bacterial lipopolysaccharide (LPS) from the involuting mammary gland into the systemic circulation during the 8 weeks before calving [32,35]. A second, complementary prepartum exposure operates in the same window and does not require Gram-negative organisms. The dry period is an infection-susceptible interval rather than a microbiologically quiescent one: among quarters bacteriologically negative at dry-off, new intramammary infection (IMI) arises in roughly 12–18% of previously healthy quarters or cows, with herd-level quarter incidence reaching 39% [40,41,42]. Because non-aureus staphylococci and environmental streptococci account for a large share of these new infections, Gram-positive cell-wall ligands and tissue injury can engage the same epithelial and leukocyte cytokine network as LPS. The hypothesis advanced here is therefore LPS-centered but not LPS-exclusive: mammary pathogen sensing and cytokine release constitute the unifying prepartum event, with endotoxin the most likely agonist during involution-associated Gram-negative exposure and a tractable experimental probe of the pathway. Experimental support for the biological plausibility of this mammary-to-liver signaling axis exists: an intramammary LPS challenge in peripartal cows is sufficient to alter systemic acute-phase indices, hepatic inflammometabolic gene expression, and liver lipid metabolism [43], although whether those hepatic effects are mediated by translocated LPS itself, by mammary-derived cytokines, or by both was not resolved, and an acute experimental bolus does not by itself establish natural translocation during dry-off. Two further experiments define the cytokine limb of this axis independently of endotoxin. A single intramammary infusion of recombinant bovine IL-8 at dry-off produced local mammary inflammation together with a systemic febrile response, showing that a mammary cytokine signal alone can act beyond the gland [44]. In experimental Staphylococcus aureus subclinical mastitis, milk mammary-associated SAA3 and haptoglobin rose within 12–15 h and preceded the serum rise at 24 h, with both mammary and hepatic acute-phase transcripts increased by 48 h [45]—precisely the mammary-to-hepatic sequence that the prepartum acute-phase findings reported here would require. Proof-of-principle evidence for organ-to-organ movement of LPS-associated material exists in goats. After intrauterine LPS administration in dexamethasone-treated animals, LPS-reactive material is detectable immunohistochemically in mammary connective tissue and alveolar spaces [46], although immunohistochemistry demonstrates LPS-reactive antigen rather than necessarily intact, biologically active LPS, and pharmacological dexamethasone is not equivalent to the endogenous cortisol elevation of parturition. The finding raises, but does not test, the hypothesis that the immunoregulatory and barrier changes accompanying calving increase interorgan exposure in transition cows. Kinetic support comes from tracer experiments in which intrauterine LPS co-infused with ink particles appeared in blood and milk within 2 h [47], demonstrating movement of uterine material into the systemic and mammary compartments, though not by itself that the detected particles were intact LPS; repeated intrauterine LPS infusion induces overt mammary inflammation with elevated plasma LBP [48]. Taken together with the intramammary-challenge data above, these studies support bidirectional functional communication among uterine, mammary, hepatic, and systemic compartments, although direct LPS-associated trafficking has been demonstrated principally in the uterus-to-mammary direction, whereas the mammary-to-liver arm rests on functional responses rather than traced endotoxin. This communicating-network architecture, rather than physiologically isolated organ compartments, is the connectivity the present hypothesis requires, and it provides a mechanistic route by which uterine and mammary disease can co-occur within one syndrome. It must be emphasized, however, that none of the studies re-analyzed here measured LPS in the mammary gland, in mammary secretions, or in blood during the dry-off period, and that the experimental models cited above used acute, high-dose challenges (and, in the goat translocation model, pharmacological dexamethasone) that are not equivalent to the low-grade, sustained exposure proposed for physiological involution. Mammary-to-systemic LPS translocation at dry-off is therefore a working hypothesis to be tested, not an established observation, and statements about a mammary source throughout this paper should be read in that conditional sense.

On current evidence, no other comparably scaled systemic LPS source has been documented during this window, although smaller uterine, intestinal, and peripheral contributions cannot be excluded. The dry-cow ration is forage-dominant, primarily hay, grass, and silage with minimal grain inclusion (the close-up ration in the cohort analyzed here contained approximately 30% concentrate, below the threshold associated with subacute ruminal acidosis [SARA] [49,50]; because rolled barley made up only 39.6% of that concentrate, cereal grain—the fraction that drives ruminal endotoxin release—supplied only about 12% of ration dry matter [6]). Under such forage-based feeding the rumen pH remains near neutral, the Gram-negative subpopulation does not expand and lyse en masse, and gastrointestinal endotoxin is not expected to enter systemic circulation in an appreciable quantity [49], although this was not measured in the present cohort. The contrast with concentrate-rich feeding is quantitative and large. Acute grain engorgement in hay-adapted cattle raised free ruminal endotoxin roughly 15- to 18-fold within 12 h [51], and controlled experiments in dairy cows report increases of the same order: feeding 45% barley grain raised ruminal endotoxin from 654 to 8870 ng/mL, approximately 14-fold, with concurrent elevation of plasma serum amyloid A, LBP, and C-reactive protein [52], and a wheat–barley subacute ruminal acidosis challenge raised rumen LPS from 10,405 to 168,391 EU/mL, approximately 16-fold [53]. Two features of this literature bear directly on the present argument. First, translocation is demonstrable but modest and conditional: a grain-based challenge raised peripheral plasma LPS from below the 0.05 EU/mL detection limit to 0.52 EU/mL, peaking at 0.81 EU/mL, together with serum amyloid A, haptoglobin, and LBP [54], whereas the same challenge in non-lactating cows left plasma LPS undetectable despite a 16-fold ruminal rise [53], the authors attributing this to hepatic clearance of translocated LPS rather than to absence of translocation. Second, the hindgut is implicated as much as the rumen: cecal LPS rose from 16,508 to 118,522 EU/g under the grain challenge but not under an iso-acidotic alfalfa-pellet challenge [53], and acidic pH combined with *Escherichia coli* LPS increases the permeability of both rumen and colon mucosa six- and fivefold, respectively [55]. Critically, however, every one of these models involves a concentrate-rich ration; they therefore characterize a postpartum exposure and cannot account for a signature already present at −8 wk on a forage-based far-off ration. Gut-barrier compromise from starch loading is similarly not operative because the substrate that drives it is not present [50]. The single exception is lameness: a cow already lame before calving, particularly with chronic digital dermatitis or a pre-existing sole lesion, may carry a peripheral Gram-negative inflammatory focus capable of contributing to systemic endotoxin load even on a forage-based diet. In the five other peripartum disease categories, mammary involution is proposed here as the principal candidate systemic LPS source during the dry-off and close-up periods, a proposal that must be weighed against the gut-origin and non-LPS alternatives considered in Section 6.7.

The situation changes after parturition. Typical early-lactation total mixed rations (TMRs) contain approximately 45–60% concentrate (grain-based), an inclusion rate that supports the rapid lactational rise in energy demand but also creates the conditions for SARA, Gram-negative die-off in the rumen, and LPS translocation across the gastrointestinal epithelium into portal and systemic circulation [32,49,50,54,56]. Consistent with this interorgan coupling, intramammary LPS challenge in dairy cows elevates systemic innate-immune and acute-phase indices [57] and alters the plasma metabolome [58]; it depresses circulating calcium and raises cortisol, with the hypocalcemia more severe in early than in mid-lactation [59]—an observation directly relevant to the hypocalcemia re-interpretation advanced earlier [36]—and the milk microbial community is restructured during mastitis and its resolution [60]. These challenge studies, from independent laboratories, do not directly trace ruminal LPS, bacteria, or metabolites into the mammary gland; what they support is functional metabolic-mammary coupling: the cow’s metabolic state conditions the local and systemic response to a mammary inflammatory stimulus—hyperketonemia, for example, reshapes both systemic and intramammary metabolism during LPS-induced mastitis [61]—and a mammary stimulus in turn has systemic metabolic consequences. Critically, the postpartum window is not a single-source environment: three microbial-ligand sources operate concurrently after calving. First, mammary involvement does not stop at calving: colostrogenesis and the abrupt onset of full lactation continue to stress the epithelium, and new or persisting intramammary infection supplies both LPS and, in the frequent Gram-positive infections, lipoteichoic acid (LTA) and other non-LPS ligands [34,40,41,42]. Second, rumen-derived LPS crosses the acidotic rumen wall as described above. Third, and quantitatively the most prominent, the postpartum uterus becomes a major endotoxin reservoir: stagnating lochia is an excellent growth medium for *Escherichia coli* and other coliforms, and uterine LPS concentrations immediately after calving average 2.24 × 10^4^ EU/mL in cows with retained placenta versus 0.26 EU/mL in healthy cows [62], with uterine-fluid endotoxin scaling with the severity of puerperal endometritis and detectable concurrently in plasma [63], and a clinical case of bovine metritis documented LPS simultaneously in plasma (0.94 EU/mL), uterine fluid (6.34 EU/mL), and ovarian follicular fluid, one follicle reaching 12.40 EU/mL, placing the ligand in the reproductive compartment at the same time as the systemic circulation [64]. Endotoxin absorbed from the uterine lumen therefore reaches the systemic circulation at the same time as the mammary and ruminal contributions. The three sources are not mutually exclusive postpartum, and the temporal architecture of the urine metabolomic signature documented here (peak cross-disease coherence at −4 wk, partial divergence at clinical disease and at +4 wk) is consistent with this changing source structure: a clean, mammary-dominant signature builds through the dry-off period, then dilutes and shifts as rumen-derived LPS and individual organ failures both contribute after calving.

LPS itself is the prototypical TLR4 ligand. Sustained low-grade TLR4 activation drives a stereotyped metabolic-immune program characterized in the broader inflammation and metaflammation literature: NF-κB-driven pro-inflammatory cytokine release (TNF-α, IL-6, IL-1β); HIF-1α-driven aerobic glycolysis (the Warburg-like metabolic shift of activated macrophages) [65,66]; IDO1 induction with consequent tryptophan depletion and kynurenine elevation [67,68,69]; mTORC1 activation driven by elevated branched-chain amino acids; phospholipase A_2_ activation with consequent lysophosphatidylcholine release and membrane lipid remodeling [70]; and a hepatic acute-phase response (haptoglobin, serum amyloid A, LPS-binding protein) [32,71,72]. Recent work in dairy cows confirms that peripheral blood mononuclear cell (PBMC) tryptophan–kynurenine pathway expression segregates healthy from diseased peripartum cows [73], placing the present cross-disease metabolomic signature on a known, conserved cellular axis. If a low-grade LPS exposure of mammary origin is sufficient to drive this program in transition cows, then every cow that subsequently develops any of the six peripartum diseases—regardless of which clinical syndrome eventually appears—should display a conserved metabolic-immune signature in the weeks before calving.

### 1.4. From Hypothesis to Testable Predictions

The single-syndrome hypothesis makes four specific predictions, each testable with a sufficiently broad metabolomic dataset.

**Prediction 1: cross-disease metabolic similarity.** If the six diseases share an upstream trigger, then disease-vs-control effect-size vectors should be positively correlated between every pair of diseases. The strength of this correlation, measured across the full metabolite panel, becomes a direct quantitative test of the syndrome hypothesis: r ≈ 0 would refute it; r ≈ 1 would support an essentially identical host response.

**Prediction 2: a conserved molecular signature.** A subset of metabolites should be altered in the same direction in all six diseases at the same preclinical timepoint. The functional content of this signature should map onto the canonical TLR4 → cytokine → IDO/HIF-1α/mTORC1/PLA_2_ downstream program [65,67,68,69,70] rather than onto disease-specific pathways.

**Prediction 3: a biphasic temporal architecture.** The conserved cross-disease signature should strengthen through the dry-off period (when mammary involution is proposed to be the dominant LPS source), peak shortly before clinical disease, partially diverge at the moment of disease as individual organ failures manifest and the single dominant prepartum source gives way to three concurrent postpartum ones (mammary, ruminal, and uterine), and then reconverge, without the magnitude of deviation from controls returning to the control state. This biphasic architecture—clean mammary-dominant signal prepartum, mixed signal postpartum—is the temporal pattern expected under the mammary-origin hypothesis and is less readily predicted by either a generic stress framework or a rumen-LPS-only framework.

**Prediction 4: convergent evidence across multiple data layers, with milk providing source-organ-informative evidence.** A single-syndrome trigger should leave its signature in every biofluid examined: serum (systemic metabolic readout), urine (renal-cleared metabolic and microbial-derived metabolites), and milk (mammary epithelial output and its mineral composition). Because milk is the secretion of the candidate source organ itself, the milk findings are particularly diagnostic: a TLR4-pattern shift in milk would directly implicate the mammary gland in the syndrome rather than place it merely on the receiving end of systemic inflammation.

### 1.5. Scope of This Paper

The peripartum metabolomic dataset accumulated over the past decade—comprising serum DI-LC-MS/MS, serum GC-MS, urinary ^1^H-NMR, milk ^1^H-NMR, milk DI-LC-MS/MS, milk ICP-MS minerals, and an innate-immunity/acute-phase/metabolic-stress panel—provides the empirical basis to test these four predictions. Most of the disease-specific findings within each matrix have been reported previously in the disease-specific literature [1,2,3,4,5,6,7,8,9,10,11,12,13,14,15,16,17,18,19,20,21,22,23,24,25,26], and the data layers that have not been published before are identified in Section 2.1; the integrative cross-disease analyses presented here have not been reported and could not, by design, have been generated by any single-disease study. These analyses are used here to evaluate whether the four predictions are met, and the resulting picture is discussed in relation to the broader inflammation and metaflammation literature [32,34,35,74,75], including direct cross-species inference to human postpartum, peri-operative, and chronic-inflammatory disease.

It is not claimed here that the data presented prove the LPS hypothesis. Direct LPS quantification—by 3-hydroxymyristic acid mass spectrometry in serum and in mammary lavage during the dry-off transition [76,77]—remains the necessary confirmatory experiment, as do mammary microbiome characterization and intervention studies that directly target mammary involution. The more modest claim is this: the existing data provide varying degrees of support for the four predictions of the LPS-driven syndrome hypothesis about the host metabolic-immune response, identifying a shared metabolic-inflammatory component across the six clinical presentations without by themselves establishing a single etiology, an LPS trigger, or its anatomical source; the predicted biphasic temporal architecture is observed; comorbidity is reframed from confounder to expected phenotype; and the framework opens specific, falsifiable next experiments that no disease-specific framework would have suggested.

## 2. Materials and Methods

### 2.1. Study Design and Origin of the Data

This hypothesis paper is an integrative, secondary re-analysis of metabolomic, immunological, and production data generated previously in the author’s laboratory at the University of Alberta. Most of these data have been published disease-by-disease (source studies listed in Table 1), but some have not (see the end of this section). No new animals were enrolled and no new samples were collected. All values shown in the figures and tables of this paper, including Table 2, are original data of the author’s laboratory: they were recomputed for the present analysis from the per-cow measurements or per-disease result tables underlying the cited source studies, and none was extracted from publications of other research groups. Literature from other laboratories is used only for mechanistic interpretation and is cited where it appears. The cross-disease analyses themselves (cross-disease correlation, concordance, permutation and resampling analyses, and time-course comparisons) have not been reported previously; they were performed in Python 3.13 and MetaboAnalyst 6.0 [78] as described in Section 2.6. The innate-immunity panel has been published for all six diseases [6,12,14,18,21,79], whereas the metabolomic layers have been published only in part [2,3,4,7,8,9,10,11,13,15,17,19,20,22,23,24]. The unpublished layers, reported here for the first time, are serum GC-MS for ketosis and milk fever; urine ^1^H-NMR for milk fever, retained placenta, and subclinical mastitis; milk ^1^H-NMR and DI-LC-MS/MS for all diseases except lameness; and all milk ICP-MS data.

### 2.2. Animals, Location, Study Period, Housing, and Diets

The source studies were nested case–control analyses within a prospective cohort of 100 pregnant multiparous Holstein cows followed from 8 weeks before to 8 weeks after expected calving at the Dairy Research and Technology Centre of the University of Alberta (Edmonton, Alberta, Canada) [6,18,21]. The project ran from 2011 to 2016: cows were enrolled and sampled in 2011–2012 (calvings between May 2011 and September 2012), after which the samples were processed and analyzed by metabolomics and the data were processed statistically. Cows were housed in individual tie stalls bedded with sawdust, with free access to water, and were moved to a maternity pen approximately one week before expected calving [6,18]. Cows were dried off according to the standard operating procedure of the Dairy Research and Technology Centre (SOP DRTC M-5.0, Initiation of Dry-off Period): milking was stopped abruptly, without intermittent milking, 60 ± 3 days before expected calving, or earlier if milk yield fell below 11 kg/d. At dry-off, each quarter received an intramammary dry-cow antibiotic followed by an internal teat sealant, teats were dipped with a post-milking teat dip, and each cow was injected subcutaneously with 5 mL of an *Escherichia coli* J-5 bacterin (Enviracor J-5, Zoetis Canada Inc., Kirkland, QC, Canada). Dry cows were then moved to an outdoor pen with other dry cows and bred heifers and were checked daily for one week to confirm that milk secretion had stopped and that they were eating normally. Rations were fed as total mixed rations once daily at 08:00 for ad libitum intake (approximately 5% orts) and were formulated to meet or exceed NRC (2001) requirements [6]. On a dry-matter basis, the close-up ration contained 60.0% barley silage, 10.0% alfalfa hay, and 30.0% close-up concentrate, and the early-lactation ration contained 30.2% barley silage, 9.6% alfalfa hay, 9.6% alfalfa silage, and 50.5% lactation concentrate [6]. As reported in the source studies, all cows received this prepartum (close-up) ration throughout the dry period and were switched gradually to the lactation ration over the first 7 days after calving [6,22,79]. In the close-up concentrate, rolled barley made up 39.6% and ground soybean hulls and canola meal most of the remainder, so cereal grain supplied only about 12% of ration dry matter. The early-lactation concentrate (50.5% of ration dry matter) contained 39.9% rolled and 27.5% ground barley, 10.0% wheat distillers grains, 8.0% high-bypass soybean meal, 3.5% blood meal, 0.5% poultry tallow, and 0.33% rumen-protected lysine, so barley supplied about 34% of ration dry matter. The full composition of both rations is given in Supplementary Table S1 [6]. Because all disease groups and their controls were drawn from the same herd, housing, rations, and management over the same period, systematic differences between the source studies in farm, diet, or dry-off protocol are unlikely to account for the cross-disease similarity; conversely, the single-herd design limits generalization to other herds, breeds, and management systems (Section 6.8). The ketosis innate-immunity data derive from a parallel investigation in the same herd with the same control design [79]. Two subclinical mastitis studies cited for context [25,26] were conducted in a separate commercial herd in Alberta and were not pooled into the cross-disease analyses. All procedures in the source studies were approved by the University of Alberta Animal Care and Use Committee: Livestock (protocol 146/02/13, “Identification of biomarkers associated with the onset and progression of major metabolic and infectious diseases of transition dairy cows”) and followed the guidelines of the Canadian Council on Animal Care [80].

### 2.3. Case Definitions, Control Selection, and Comorbidity

Cows were monitored daily, and cases were diagnosed using the definitions of the source studies: ketosis, serum β-hydroxybutyrate ≥ 1.4 mmol/L together with a urine Ketostix (Bayer Inc., Toronto, ON, Canada) reading above “moderate” (3.92 mmol/L acetoacetate) [79]; lameness, locomotion score 5 on a five-point scale, compared with controls scoring 1 [6]; milk fever, clinical parturient paresis (stage 3; recumbency) diagnosed by a veterinarian within 24–48 h after calving on the basis of clinical signs and response to calcium treatment [12,13]; metritis, reddish-brown fetid vaginal discharge with fever (rectal temperature > 39.5 °C), an abnormally large uterus, and decreased feed intake and milk yield within 3 weeks after calving [14]; retained placenta, failure to expel the fetal membranes within 24 h after calving [18]; and subclinical mastitis, somatic cell count > 200,000 cells/mL on at least two consecutive tests without visible abnormalities of milk or udder [21]. Controls were cows from the same cohort that remained free of all recorded peripartum diseases during the study; in the innate-immunity studies they were matched to cases by parity and body condition score [6,18,21]. Because all prepartum samples were collected before any diagnosis, cases and controls were assigned retrospectively. The source studies retained only cows with a single diagnosis and excluded cows with concurrent peripartum diseases [2,3,4,7,8,9,10,11,13,14,15,17,18,19,22,23,24,79]. The exception relevant to the present analyses is the per-cow serum DI-LC-MS/MS file, in which the six cows carrying the ketosis label also appear in other disease groups (four with identical metabolite profiles); in that file the ketosis label is therefore a comorbid-composite label rather than an independent group (Section 3.2). Its influence was assessed by repeating the similarity analyses on the five non-overlapping disease groups (Section 2.6).

### 2.4. Sampling Schedule and Analytical Platforms

Blood, urine, and fecal samples were collected weekly, before the morning feeding, from −8 wk before expected calving to +8 wk after calving, and milk samples were collected weekly during weeks 1–8 of lactation. The analyses used blood and urine from −8 and −4 wk before expected calving, the week of disease diagnosis, and +4 and +8 wk after calving, and milk from +2 wk. The diagnosis-week sample fell at different intervals after calving in different diseases (for example, within 48 h for milk fever and 1–3 weeks postpartum for ketosis and lameness) [4,9,13]. The data layers were: serum targeted DI-LC-MS/MS (128 metabolites retained after quality control); serum GC-MS (40 metabolites); urine ^1^H-NMR (91 metabolites); milk ^1^H-NMR (36 metabolites); milk DI-LC-MS/MS (124 metabolites); milk ICP-MS (18 elements, of which arsenic was measured only in the retained placenta study); and a serum panel of nine innate-immunity, acute-phase, and metabolic-stress markers (IL-1, IL-6, TNF-α, haptoglobin, serum amyloid A, LPS-binding protein, lactate, non-esterified fatty acids, and β-hydroxybutyrate) measured with commercial immunoassay and colorimetric kits [6,12,14,18,21,79]. Sample collection and storage, extraction, instrument settings, and quality control are described in the source studies listed in Table 1.

### 2.5. Source Studies and Sample Sizes

The published studies from the author’s laboratory that supplied data for this analysis are listed in Table 1, with the biological matrix, analytical platform, sampling timepoints, and numbers of case and control cows of each. Except for the two commercial-herd studies [25,26], all were nested case–control studies within the same Dairy Research and Technology Centre cohort. In these studies, disease groups were small (six cows per disease; four milk fever cows after calving [13]), and, within each study, all cases were compared with one control group. The unpublished data layers listed in Section 2.1 were generated in the same cohort with the same design: six cases for each of the six diseases (36 cases in total) and the same 20 healthy control cows, which served as the common control group for all six diseases.

### 2.6. Data Processing and Statistical Analysis

Pre-processing followed the source studies: values that were missing or below the limit of detection were replaced by one-half of the minimum positive value of the respective metabolite [4,13,20], and urinary concentrations were normalized to creatinine [4,9,23]; milk concentrations were log-transformed and autoscaled before multivariate analysis [11]. No further imputation was performed in the integrative analysis, and a metabolite was included in a matrix only if it was quantified in all disease groups of that matrix (the common metabolite set). For each disease and timepoint, the effect size of each metabolite was calculated as log2FC = log_2_(mean_disease_/mean_control_), using arithmetic group means of the pre-processed concentrations and the contemporaneous controls of the same matrix and timepoint (for the serum GC-MS diagnosis window, the +2 wk control sampling). Per-disease significance used the univariate test of the source study (two-sided Mann–Whitney *U* or *t* test), and Benjamini–Hochberg *q*-values were computed within each disease and timepoint. Cross-disease similarity was defined as the mean of the 15 off-diagonal Spearman rank correlations (10 for five groups) between per-disease log2FC vectors over the common metabolite set. Concordance was the number of metabolites whose log2FC had the same sign in all disease groups; where stated, *p* < 0.05 in every group was additionally required. Principal component analysis (PCA) used log_10_-transformed, autoscaled per-cow data. The kynurenine/tryptophan (Kyn/Trp) ratio was calculated per cow and compared with controls by two-sided Mann–Whitney *U*, Student’s *t*, and Welch’s *t* tests.

Four dependency-aware sensitivity analyses were applied to the per-cow serum data at −8 wk. (i) Label permutation: case and control labels were permuted 2000 times while preserving group sizes and the common control group, and the mean pairwise *r* was recomputed each time; the permutation *p*-value is the proportion of permutations that reached the observed value. (ii) Leave-one-control-out: the mean *r* was recomputed 20 times, each time omitting one control cow. (iii) Cross-fitting: for each disease pair, the control group was randomly split into two disjoint halves (*n* = 10 each), one half serving as the reference for each disease, so that the two effect-size vectors shared no control animal; this was repeated over multiple random splits, and the 95% interval is the 2.5th–97.5th percentile of the resulting means. (iv) Cow-level bootstrap: cows were resampled with replacement within each group and the mean *r* recomputed (percentile 95% confidence interval). For the five-timepoint per-disease tables (Section 4.4), a metabolite-order permutation null was used: the order of metabolites was shuffled independently within each disease vector 500 times, which preserves each disease’s effect-size distribution while destroying alignment between diseases. The magnitude of deviation from controls was summarized as the mean absolute log2FC across metabolites. Analyses were performed in Python 3.13 (pandas 3.0, NumPy 2.5, and SciPy 1.18) with a fixed random seed and in MetaboAnalyst 6.0 [78].

### 2.7. Interpretation of the Evidence

All analyses are observational and correlational, and LPS itself was not measured in any source study. Throughout this paper, a prediction is described as supported when the data agree with it and do not refute it; this wording does not imply that a causal relationship, the LPS trigger, or its mammary source has been demonstrated.

## 3. The Conserved Metabolic-Immune Signature

### 3.1. Cross-Disease Similarity Is Positive in Every Matrix and at Every Timepoint

**Figure 1:**
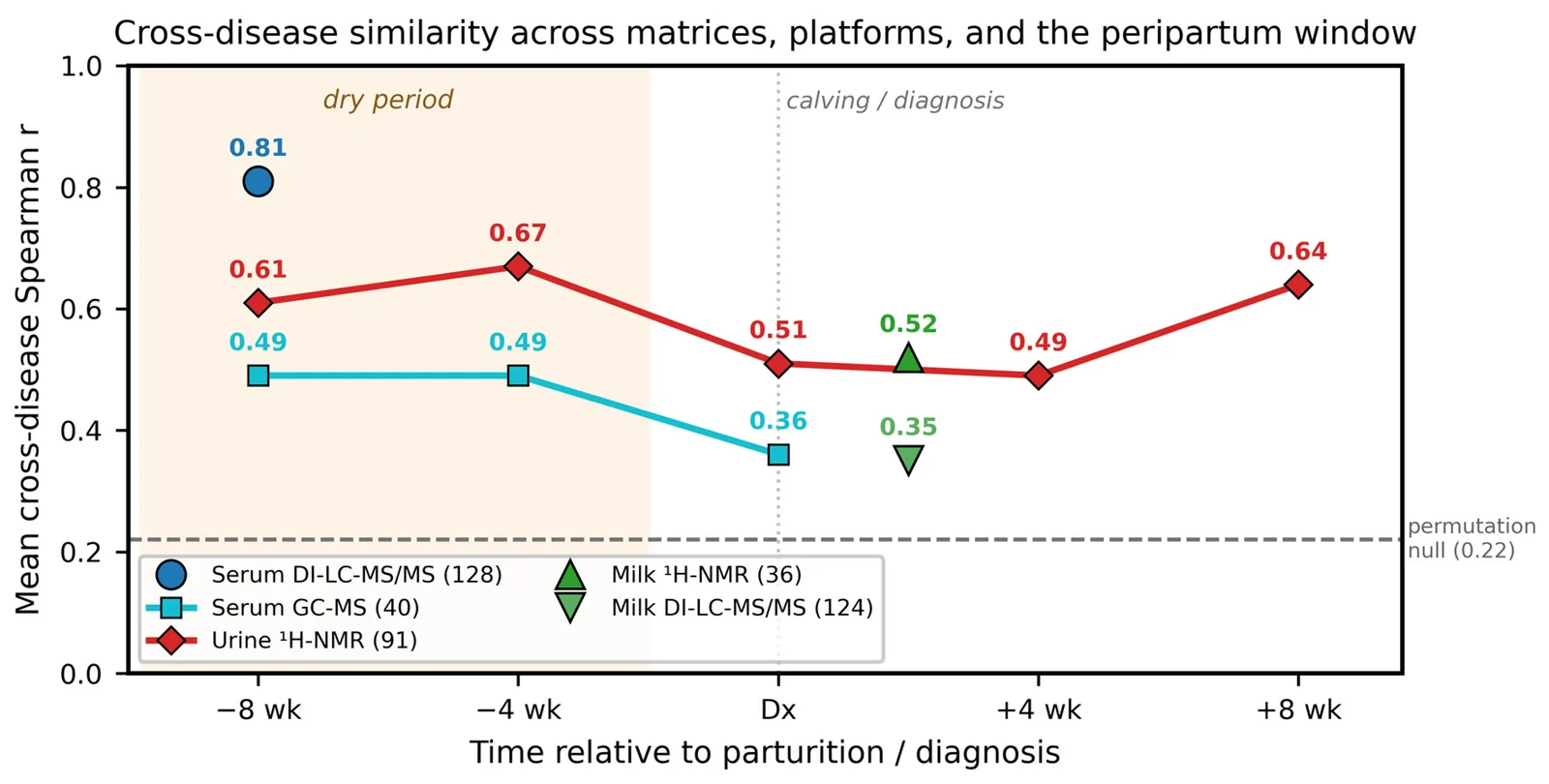
Cross-disease similarity is positive in every matrix and at every timepoint examined. Each bar represents the mean off-diagonal Spearman correlation between disease-vs-control log2 fold-change vectors across the six peripartum diseases (serum GC-MS at diagnosis: five diseases; milk fever excluded at *n* = 2). The serum DI-LC-MS/MS panel (−8 wk; 128 metabolites) shows the strongest similarity (r = 0.81). The serum GC-MS panel (40 metabolites) is positive at −8 and −4 wk (r = 0.49) and dips at diagnosis (r = 0.36). The urine NMR panel (91 metabolites) shows positive similarity at every timepoint of the peripartum window, with peak coherence at −4 wk (r = 0.67). Milk NMR (36 metabolites; +2 wk) gives r = 0.52. The milk DI-LC-MS/MS panel (124 metabolites; +2 wk) gives r = 0.35. The eleven measurements span five metabolomic data layers (milk ICP-MS minerals are evaluated separately in Section 5.2). The dashed line at r = 0.5 is an arbitrary visual reference, not a statistical threshold. All eleven values exceed what the shared-control design alone produces under permutation (Section 3.1).

The most direct test of the single-syndrome hypothesis is whether the metabolic effect-size vectors elicited by the six diseases are correlated with each other. Positive cross-disease correlation would support a shared component of metabolic variation. Because all disease contrasts are referenced to one common control group, however, the shared control term makes the effect-size vectors statistically dependent and induces positive covariance even between unrelated diseases; the observed correlations must therefore be judged against a null model that preserves that structure rather than against zero. Across the five metabolomic data layers analyzed here—serum DI-LC-MS/MS, serum GC-MS (three peripartum windows), urinary ^1^H-NMR (across five timepoints), milk ^1^H-NMR, and milk DI-LC-MS/MS, with milk ICP-MS minerals evaluated separately in Section 5.2—every cross-disease similarity measurement is positive at the mean level, with mean off-diagonal Spearman r ranging from 0.35 to 0.81 (Figure 1; metabolomic statistical analyses were performed with MetaboAnalyst [78]). A complementary serum platform corroborates the serum result: GC-MS quantification of 40 serum metabolites in the same cohort (reported disease-by-disease in [7,16,20,22]) yields uniformly positive cross-disease similarity before calving (mean r = 0.49 at both −8 and −4 wk, with 22 of 40 metabolites concordant in all six diseases at each window) that drops to r = 0.36 at clinical diagnosis—the same divergence-at-disease behavior documented longitudinally in urine (Section 4.2). (In the GC-MS diagnosis window, milk fever contributed only two cows and was excluded; the postpartum control reference is the +2 wk sampling.)

The serum measurement at −8 wk is particularly striking: every pair of the six diseases shows r between 0.72 and 0.89. By 8 weeks before clinical disease, long before any individual organ has overtly failed, the disease-state cows are converging on a common metabolic profile. This pattern is consistent with a common upstream perturbation and is less readily explained by six fully independent etiologies, although the shared herd, diet, and management and the shared control group also contribute to similarity (Section 2.2 and Section 2.6). The urine and milk measurements give numerically smaller but qualitatively identical conclusions, with no pairwise correlation dropping below r = 0.13 in any of these matrices; only in the 124-metabolite milk DI-LC-MS/MS panel does a single disease pair fall to r ≈ 0 (Section 5.1). Four dependency-aware sensitivity analyses were performed on the per-cow serum data to quantify how much of this similarity the shared-control design alone can produce. First, a label-permutation null preserving group sizes, the common control group, and the empirical metabolite covariance yields a mean pairwise r of 0.22 (95th percentile 0.44), confirming that the design does inflate correlation above zero, but far below the observed 0.81 (six labels) and 0.79 (five non-overlapping labels); both exceeded the null in every one of 2000 permutations (*p* < 0.001). Second, leave-one-control-out recomputation changes the five-group mean by less than 0.02 (range 0.78–0.80), so no single control animal drives the result. Third, cross-fitting each disease pair against disjoint halves of the control group, removing the shared denominator entirely, still gives mean r = 0.71 (95% CI 0.62–0.76). Fourth, a cow-level bootstrap gives mean r = 0.73 (95% CI 0.65–0.80). Cross-disease similarity is therefore attenuated but not abolished when the shared-control dependency is removed, and it remains well above what the design alone generates. The four predictions identified in Section 1.4 are therefore evaluated against a dataset in which the basic syndromic structure is already in evidence at the level of bulk cross-disease comparison; the remaining sections examine which metabolites carry that signal and what they imply about the mechanism.

### 3.2. Cross-Disease Similarity in Serum at −8 wk Is Uniformly High

**Figure 2:**
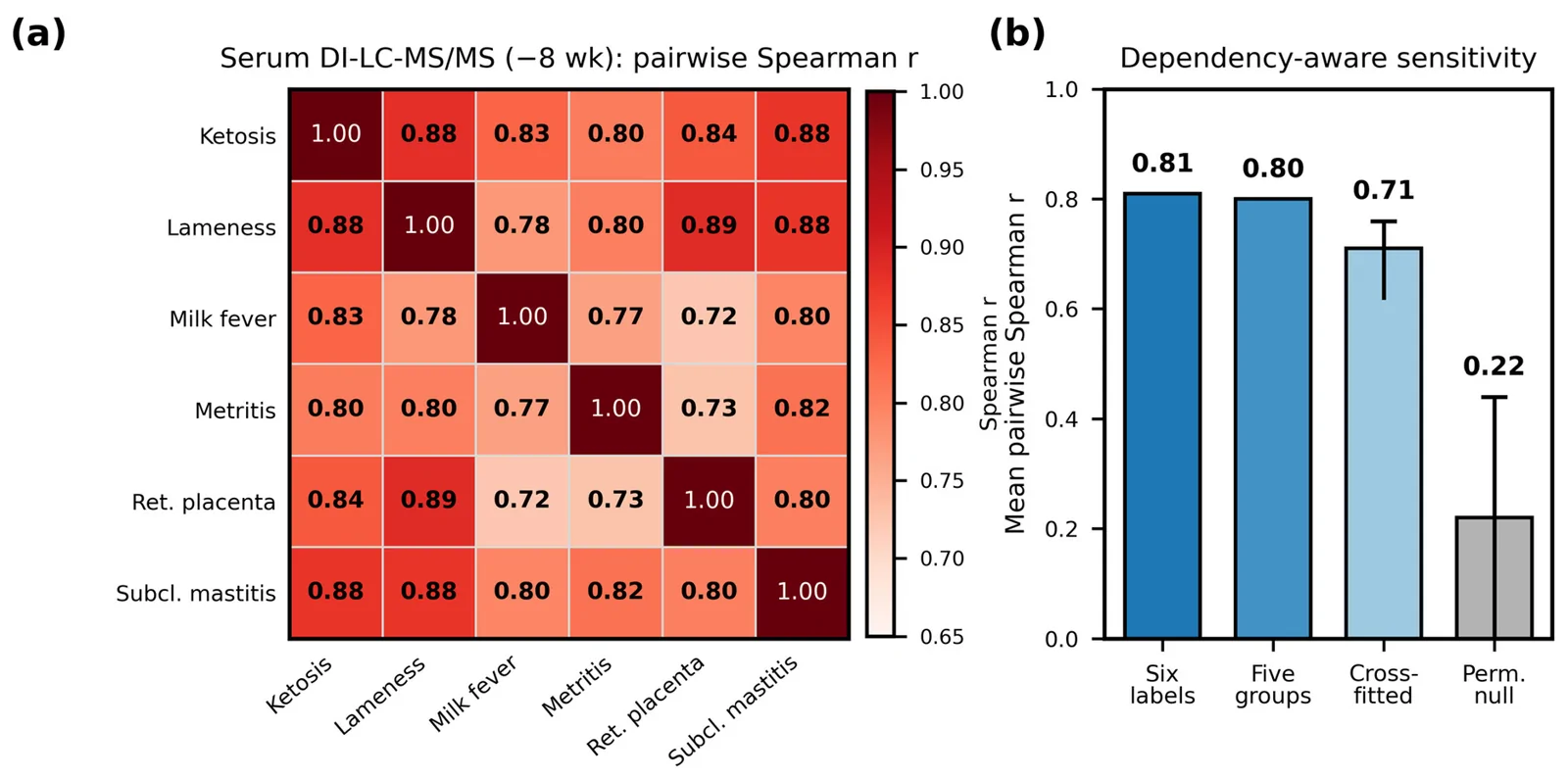

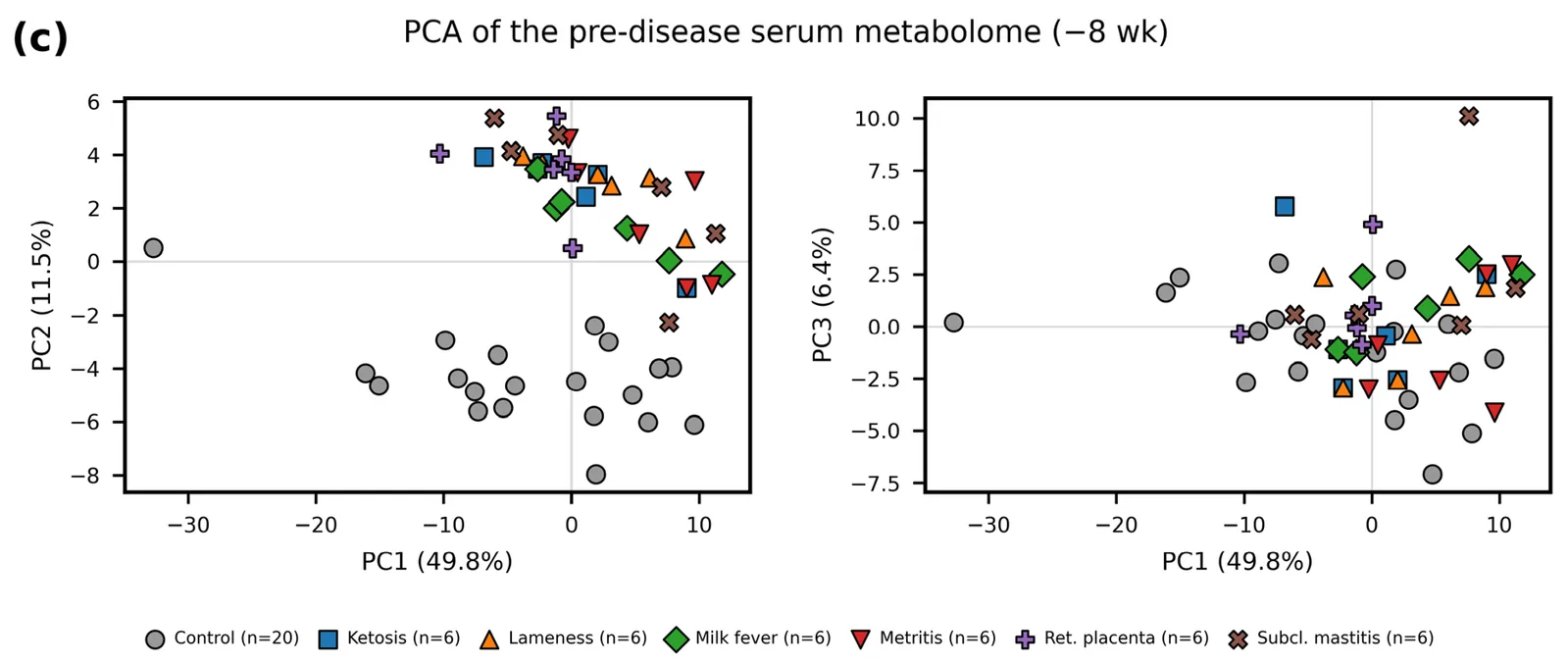
(**a**) Cross-disease Spearman correlation matrix of serum DI-LC-MS/MS log2 fold-change vectors at −8 wk pre-calving. Every pairwise correlation is positive (range 0.72–0.89; mean 0.81). The pattern is consistent with a common upstream perturbation rather than six independent etiologies. (**b**) Dependency-aware sensitivity of the mean pairwise correlation (Section 3.1). Bars show the mean pairwise Spearman r computed across the six clinical labels (0.81) and across five non-overlapping groups after collapsing comorbid animals (0.80); cross-fitting each disease pair against disjoint halves of the control group, which removes the shared-control denominator entirely, still yields 0.71 (error bar, 95% CI 0.62–0.76). The shared-control label-permutation null yields a mean of 0.22 (error bar, 95th percentile 0.44); observed values exceeded the null in all 2000 permutations (*p* < 0.001). (**c**) Principal component analysis of the serum metabolome at −8 wk (left: PC1 versus PC2; right: PC1 versus PC3). PC1 (49.8% of variance) separates control cows from disease cows. The six disease groups intermingle on the disease side, identifying a dominant shared disease-associated axis; this exploratory analysis does not by itself establish a single etiology, and comorbid animals contribute more than one label.

The serum DI-LC-MS/MS panel is the largest targeted metabolite panel analyzed here (128 metabolites covering amino acids, biogenic amines, acylcarnitines, sphingomyelins, lysophosphatidylcholines, and glycerophosphocholines). Pairwise Spearman correlations between the six disease-vs-control log2 fold-change vectors are presented in Figure 2a. All fifteen off-diagonal values are positive, ranging from r = 0.72 (milk fever vs. retained placenta) to r = 0.89 (lameness vs. retained placenta); the mean is 0.81. The dependency-aware sensitivity analyses described in Section 3.1, which show that this mean is attenuated but not abolished when the shared-control dependency is removed, are summarized in Figure 2b. Principal component analysis on the same data (Figure 2c) recovers a single dominant axis (PC1, 49.8% of variance) that separates control cows from disease cows, with the six disease groups intermingling on the disease side of PC1 rather than each occupying a distinct region.

The PCA result deserves emphasis. If the six diseases were driven entirely by distinct, non-overlapping processes, some separation among disease groups would be expected in the dominant components. Instead, PC1 captures a single direction of biological variation that all six disease groups share, and PC2 (explaining far less variance) does not cleanly separate the disease groups from each other either. By 8 weeks before clinical diagnosis, a dominant shared disease-associated axis is already present. This exploratory analysis supports a common component of metabolic variation but does not by itself establish a single upstream etiology or exclude disease-specific mechanisms; comorbid animals also contribute more than one label (Section 3.2). This is the cross-sectional analog of the longitudinal trajectory shown later in Section 4.2. A cohort-structure audit of the underlying per-cow serum file adds an important qualification: the six cows carrying the ketosis label at −8 wk are the same animals that appear in the other disease groups (four with identical metabolite profiles); in this file, the ketosis group is a comorbid-composite label rather than an independent sixth cohort. Excluding it leaves five non-overlapping disease groups and changes the mean −8 wk similarity only from r = 0.81 to r = 0.80 (range 0.72–0.89): the cross-disease convergence is not explained by shared animals. That the comorbid-composite label carries the same conserved signature is also consistent with the phenotype the syndrome predicts: cows that exit through one clinical door frequently exit through another.

### 3.3. Thirteen Serum Metabolites Are Concordantly Altered in All Six Diseases

**Figure 3:**
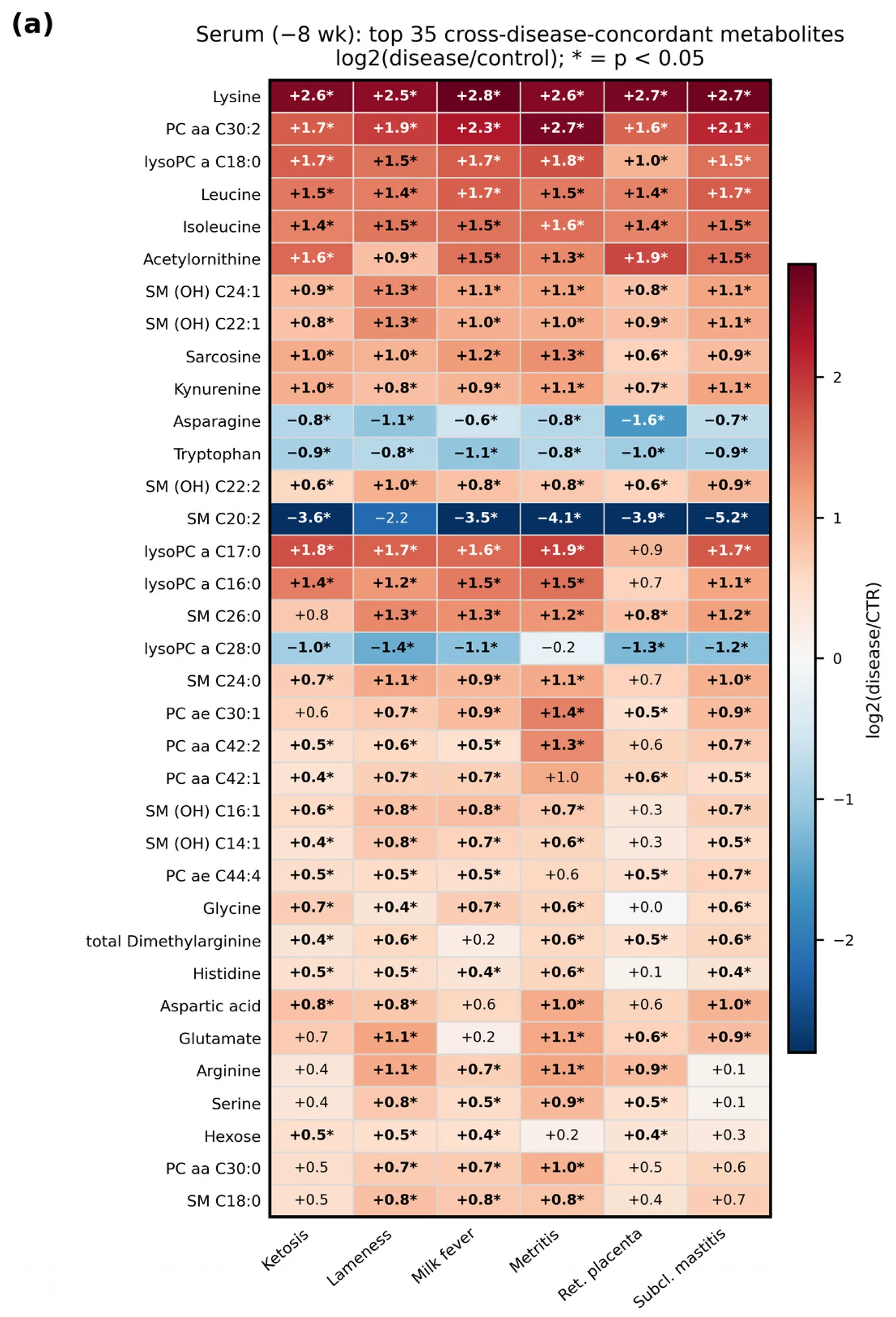

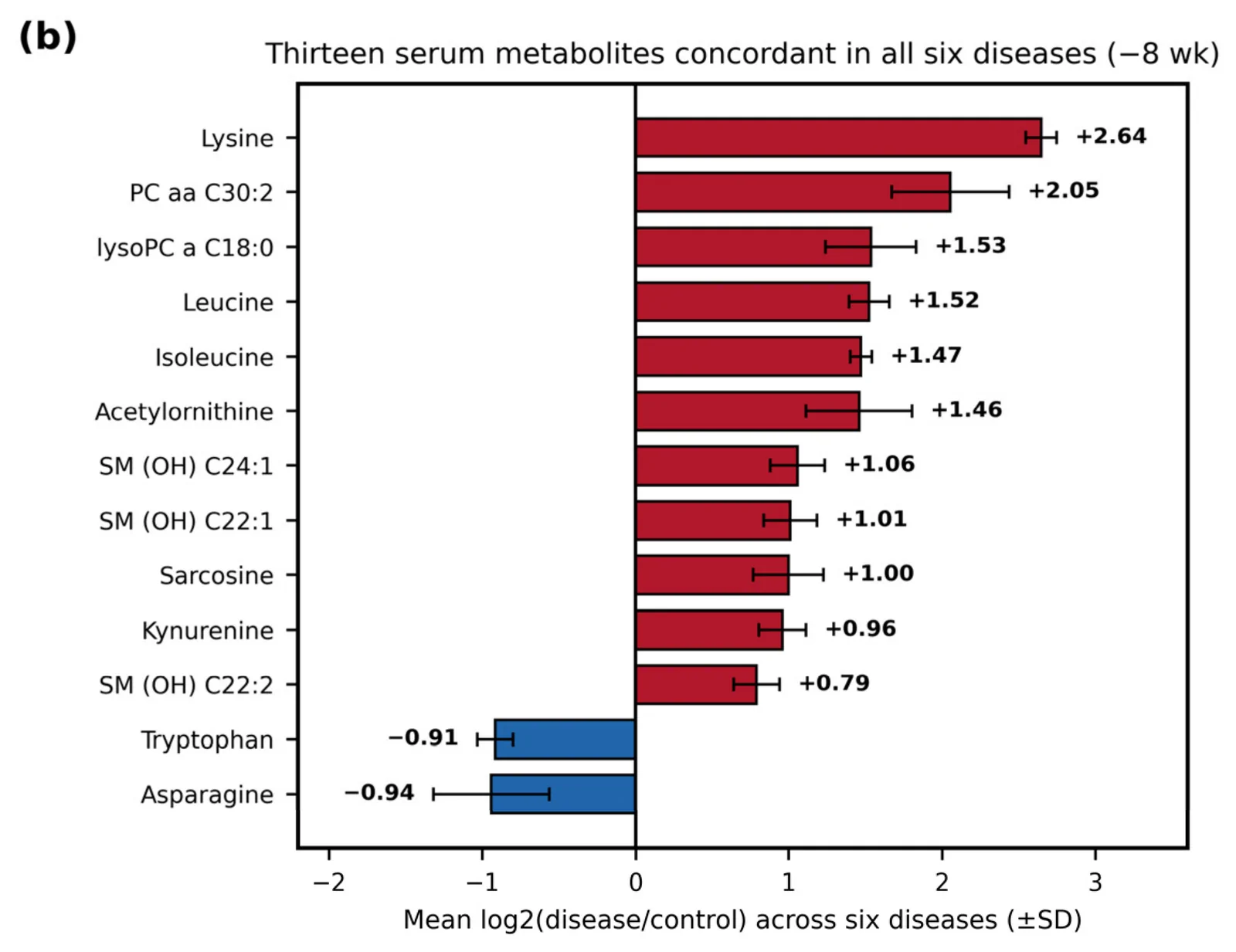
(**a**) Heatmap of the top 35 most cross-disease-concordant serum metabolites at −8 wk. Rows are metabolites ordered by concordance across the six diseases; columns are diseases. Color encodes log2(disease/control), with red indicating higher and blue indicating lower concentrations in disease than in controls; values in bold with asterisks denote *p* < 0.05. Thirteen metabolites are concordant in all six diseases (concordance score = 6). (**b**) The thirteen serum metabolites altered in the same direction in all six peripartum diseases at −8 wk. Mean log2 fold change across diseases is shown; red bars indicate metabolites that are higher and blue bars metabolites that are lower in disease than in controls, and error bars show the SD across the six diseases. Functional grouping: branched-chain and substrate amino acids (Lys, Leu, Ile, Asn); IDO axis (Trp, Kyn); polyamine pathway (Acetylornithine); one-carbon flux (Sarcosine); membrane phospholipid remodeling (lysoPC a C18:0, PC aa C30:2); sphingomyelin/ceramide pathway flux (three SM(OH) species).

A more granular question is which specific metabolites carry the cross-disease signal. After per-disease univariate analysis, thirteen metabolites are altered in the same direction with *p* < 0.05 in all six disease labels at −8 wk (Figure 3a,b, Table 2); six of them—lysine, leucine, isoleucine, tryptophan, kynurenine, and PC aa C30:2, including both members of the IDO1 axis—additionally survive within-disease false discovery rate (FDR) correction (*q* < 0.05) in all six, with the remainder doing so in two to five diseases. Several members of this signature were flagged individually in the disease-specific serum studies of this cohort—amino acid and phosphatidylcholine alterations preceding ketosis [2], metritis [15,16], retained placenta [19,20], and subclinical mastitis [22,24]—but their six-disease concordance emerges only in the present cross-disease analysis. Eleven are elevated in disease (lysine, log2FC = +2.64; PC aa C30:2, +2.05; lysoPC a C18:0, +1.53; leucine, +1.52; isoleucine, +1.47; acetylornithine, +1.46; SM(OH) C24:1, +1.06; SM(OH) C22:1, +1.01; SM(OH) C22:2, +0.79; sarcosine, +1.00; kynurenine, +0.96), and two are reduced (asparagine, −0.94; tryptophan, −0.91). Notably, this thirteen-metabolite signature is not enriched for any disease-specific pathway. Instead, every member of the signature has a documented link to TLR4/NF-κB-associated metabolism in macrophages and other innate-immune cells [65,67,68,69,70], although several members (branched-chain amino acids, the kynurenine pathway, one-carbon metabolites, and sphingolipids) also respond to glucocorticoids, nutritional state, and lipomobilization (Section 6.7).

The functional structure of the thirteen-metabolite signature is informative. Branched-chain amino acid elevation (Leu, Ile) is consistent with the substrate shift associated with mTORC1 signaling during myeloid activation, although circulating concentrations do not directly demonstrate mTORC1-driven myeloid expansion. Lysine and asparagine reflect the same substrate-supply axis: lysine supplied to immune cells, asparagine consumed by them. Acetylornithine is a polyamine-pathway intermediate that may reflect arginase-associated metabolism, though it is not uniquely an M2-macrophage marker. Sarcosine is consistent with altered one-carbon metabolism (glycine N-methyltransferase activity); concentration alone is not a direct measure of flux. The three sphingomyelin (SM(OH)) species reflect the ceramide pathway flux that accompanies membrane lipid remodeling under inflammation. The lysoPC species and PC aa C30:2 are direct products of phospholipase A_2_ acting on membrane phosphatidylcholines, which is itself activated downstream of TLR4 [70]. Finally, the kynurenine elevation paired with tryptophan depletion is the characteristic biochemical signature of indoleamine 2,3-dioxygenase 1 (IDO1) activation [67,68,69], the rate-limiting enzyme of the kynurenine pathway, induced by TNF-α and IFN-γ (which are themselves NF-κB/TLR4 outputs).

The probability that thirteen metabolites would line up in the same direction across six disease groups by chance is remote (a qualitative statement; no formal null model is implied). Because the disease groups share a control group and metabolites are intercorrelated, the thirteen concordances are not independent events, and their functional grouping is suggestive rather than a formal enrichment result. Prediction 2 (Section 1.4) is therefore supported: the signature is conserved across diseases and is compatible with the canonical TLR4 program; however, because none of these metabolites is specific to TLR4 signaling, the mapping is interpretive rather than demonstrated.

### 3.4. The Tryptophan→Kynurenine Axis Is Activated 3–4× in Every Disease

**Figure 4:**
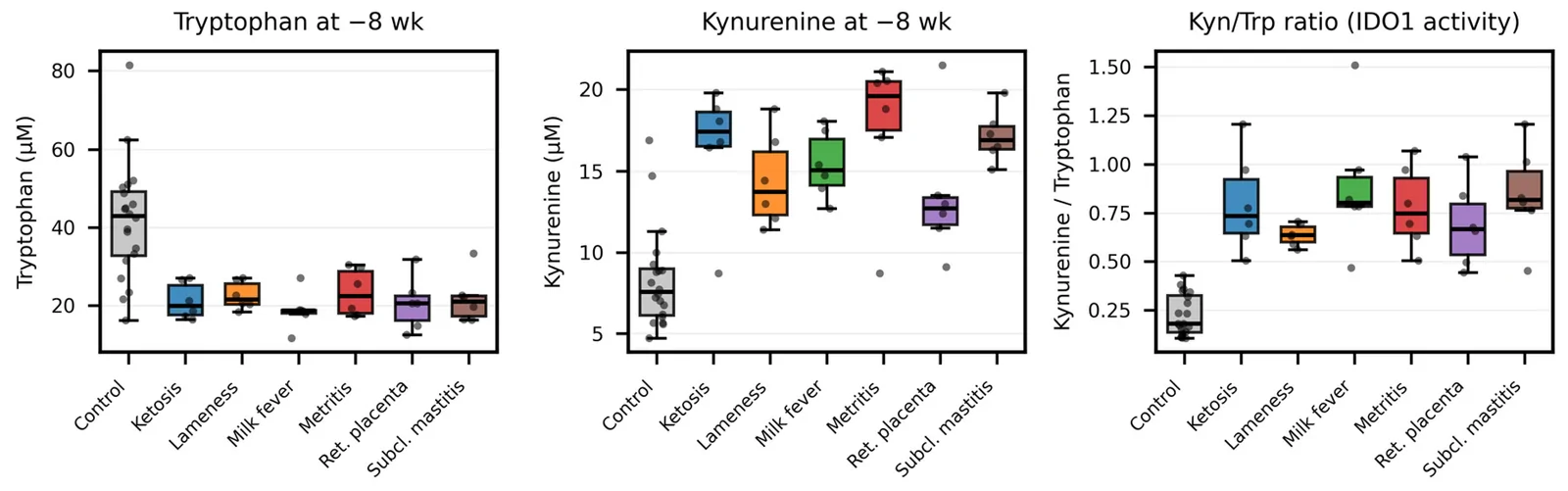
Tryptophan, kynurenine, and the kynurenine/tryptophan ratio in serum at −8 wk pre-calving across all six peripartum diseases versus control. (Left) Tryptophan is reduced in every disease. (Center) Kynurenine is elevated in every disease. (Right) The Kyn/Trp ratio is elevated 2.8–4.0× in every disease, with complete disease–control separation (all *p* = 8.7 × 10^−6^, Mann–Whitney U, the minimum attainable for these group sizes). This is the characteristic biochemical readout of IDO1 induction, the rate-limiting enzyme of the kynurenine pathway, which is induced downstream of TLR4/NF-κB by IFN-γ and TNF-α [67,68,69]. The uniform activation of this axis across all six clinically distinct peripartum diseases, quantitatively similar in magnitude, is consistent with a conserved upstream inflammatory signal. Data are original per-cow measurements from the author’s laboratory (cohort described in Section 2.2; previously reported by disease in [2,8,13,15,19,24]), re-analyzed here; controls, *n* = 20; each disease, *n* = 6.

Of the thirteen members of the conserved signature, the tryptophan–kynurenine axis warrants individual attention because it provides the single strongest mechanistic clue in the dataset (Figure 4). The kynurenine/tryptophan ratio, a ratiometric measure of IDO1 activity that is largely independent of dietary tryptophan supply, is elevated 2.8- to 4.0-fold in every one of the six diseases compared with controls. (The ratio is an index rather than a direct enzymatic assay: tryptophan 2,3-dioxygenase (TDO) activity, hepatic and renal handling, and generalized inflammation can also move it; its interpretive weight here derives from its concordance with the wider TLR4-pattern signature rather than from specificity in isolation.) The elevations are highly statistically significant: *p* = 8.7 × 10^−6^ in every disease by Mann–Whitney U test, the minimum attainable *p*-value for six diseased versus twenty control cows, reflecting complete separation of the groups: the lowest Kyn/Trp ratio observed in any disease group (0.44, retained placenta) exceeds the highest control value (0.43). Parametric testing agrees (Student’s t, *p* ≤ 5.1 × 10^−8^ in every disease; Welch’s t strongest for lameness at 9.5 × 10^−10^). The ratio is not carried by either analyte alone: in the underlying per-disease panels, kynurenine is individually elevated at *p* < 0.05 in every one of the six diseases (largest *p* = 0.0048; as low as 1.6 × 10^−9^ in subclinical mastitis), and tryptophan is individually reduced in every one (largest *p* = 0.0018).

Three features of this finding are particularly diagnostic. First, the magnitude of the effect (3–4×) is large by metabolomic standards. Second, the effect is not subtle in any disease: the fold-elevation spans 2.8× (lameness) to 4.0× (milk fever), and every disease group separates completely from controls. Third, the IDO1 axis is induced downstream of TLR4/NF-κB signaling: IFN-γ and TNF-α, among its most potent inducers, are themselves NF-κB-regulated cytokines [67,68,69], although IDO1 induction is not exclusive to TLR4 input. Recent work in dairy cows confirms that PBMCs from healthy versus diseased transition cows differ specifically in their kynurenine pathway gene expression [73]. The peripheral (serum) signature documented here is therefore on the same molecular axis as the cellular signature documented at the PBMC level by independent investigators in the same species. This convergence supports, but does not prove, involvement of the same pathway.

## 4. The Temporal Architecture of the Syndrome

### 4.1. Innate-Immunity, Acute-Phase, and Metabolic-Stress Markers Progressively Rise from −8 wk to Clinical Disease

**Figure 5:**
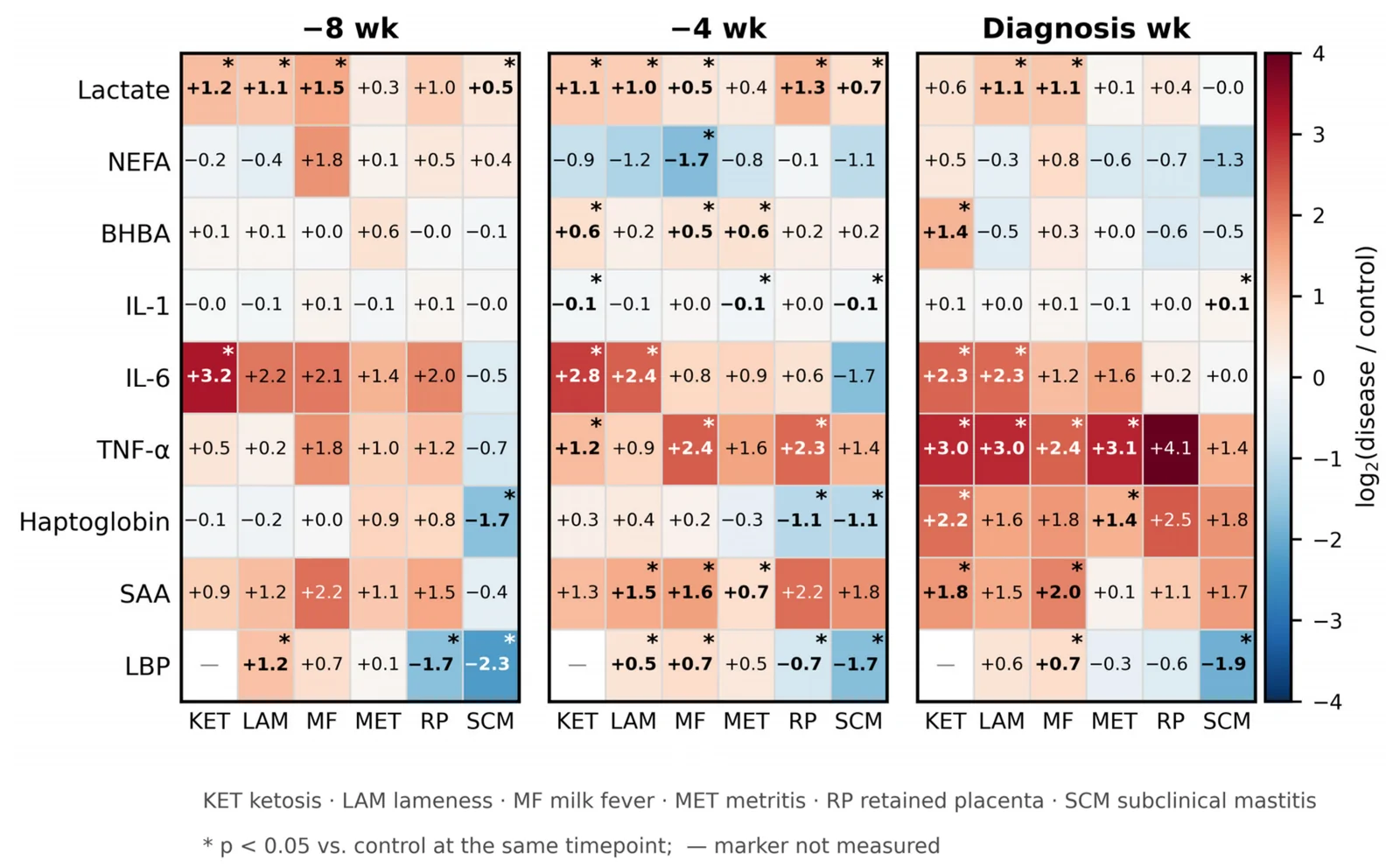
Host response across all six peripartum diseases at three preclinical timepoints. Innate-immunity, acute-phase, and metabolic-stress markers are shown as log2(disease/control) at −8 wk, −4 wk, and the week of clinical diagnosis. Color encodes the magnitude and direction of change; an asterisk in the upper-right corner of a cell denotes *p* < 0.05 versus contemporaneous controls, and an em dash denotes a marker not measured in that group. KET, ketosis; LAM, lameness; MF, milk fever; MET, metritis; RP, retained placenta; SCM, subclinical mastitis. Ketosis data are extracted from Zhang et al. 2016 [79], which used the same control cohort design as the present panel; control values match within 0.5% across the two investigations. Lactate is elevated in 6/6 diseases at −8 wk (4 significant) and 6/6 at −4 wk (5 significant), consistent with TLR4-driven aerobic glycolysis [65,66]. TNF-α, IL-6, SAA, and haptoglobin progressively rise from −8 wk to disease diagnosis, with TNF-α reaching 6/6 concordance at −4 wk and at disease diagnosis. The IL-6 signal in ketosis at −8 wk (log2FC = +3.25; ~10-fold elevation, *p* < 0.01) is particularly striking and was interpreted by the original investigators as suggestive of “an endotoxin insult” during the dry-off period [79], a speculation with which the present integrative analysis is consistent, although LPS itself was not measured. LBP shows a bimodal pattern across the five diseases in which it was measured (elevated in MF, LAM, and MET; reduced in RP and SCM); this bimodality is reframed in Section 6.3. LBP was not measured in the ketosis cohort and is shown as missing.

A panel of nine innate-immunity, acute-phase, and metabolic-stress markers (IL-1, IL-6, TNF-α, haptoglobin, serum amyloid A (SAA), LPS-binding protein (LBP), lactate, non-esterified fatty acids (NEFA), and β-hydroxybutyrate (BHBA)) was assayed at three timepoints (−8, −4, and disease diagnosis) across all six disease groups. The disease-specific components of this panel have been reported individually for lameness [6], milk fever [12], metritis [14], retained placenta [18], and subclinical mastitis [21], in each case documenting activation of innate immunity and altered carbohydrate metabolism before clinical signs; the six-disease integration is presented here for the first time. Five of the diseases were assayed within a single matched-design study; the ketosis cohort, run as part of a parallel investigation in the same herd and with the same control cohort, has been published separately [79], and the eight markers measured in that investigation (LBP excepted) are integrated here. Crucially, control values match across the two investigations to within 0.5% for every marker at every timepoint, suggesting that the cohorts are comparable. All animal procedures in the underlying studies complied with the guidelines of the Canadian Council on Animal Care [80]. The complete cross-disease pattern is shown in Figure 5.

**Figure 6:**
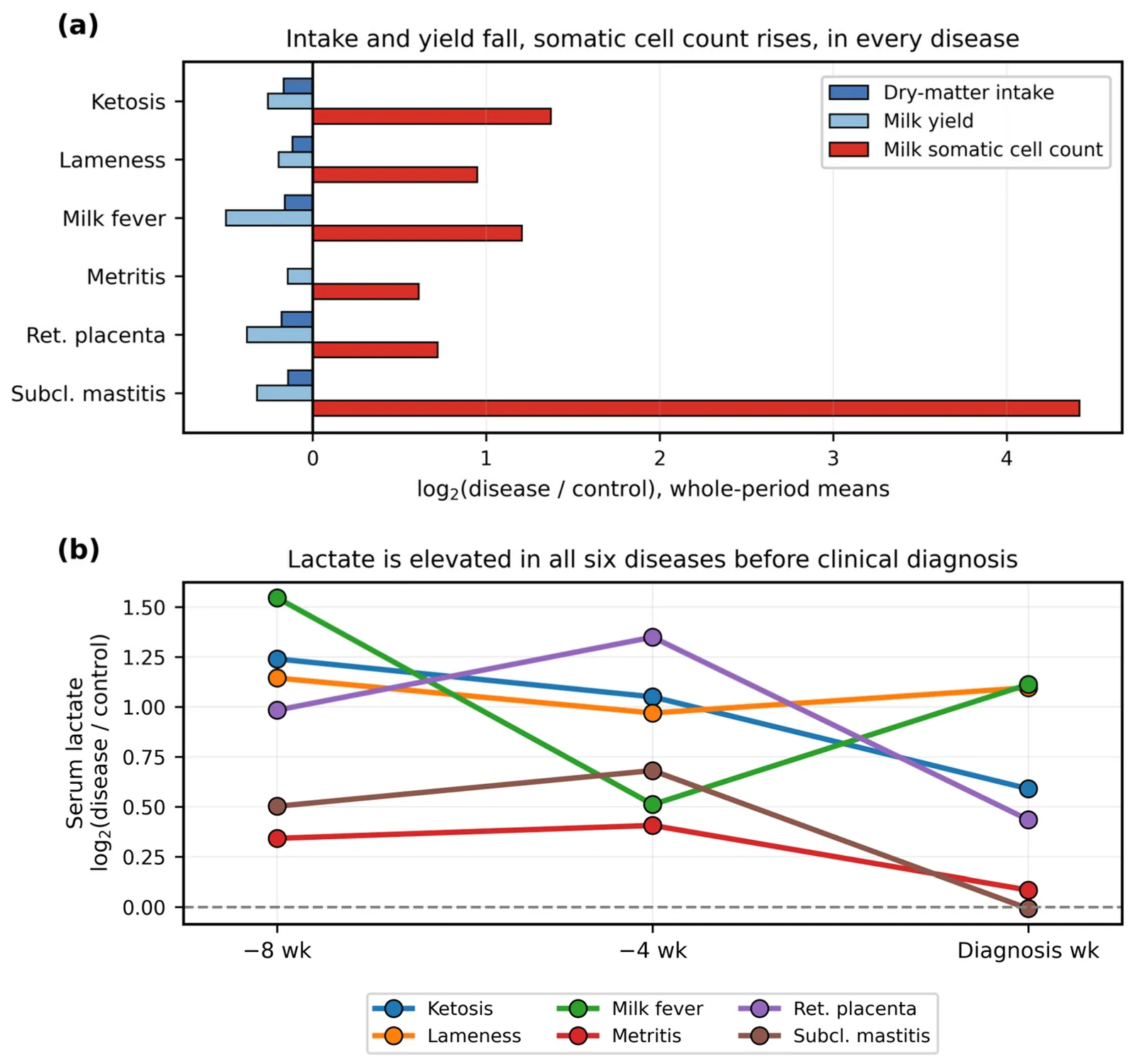
Clinical outputs and serum lactate across the six peripartum diseases. (**a**) Whole-period clinical outputs expressed as log2(disease/control): dry-matter intake and milk yield are reduced and milk somatic cell count is elevated in every disease. (**b**) Serum lactate, log2(disease/control), from −8 wk to the week of diagnosis; lactate is above control in all six diseases before any clinical sign appears, consistent with TLR4-driven aerobic glycolysis [65,66]. The dashed horizontal line marks zero, i.e., no difference from controls.

The production data recorded alongside these markers (Figure 6a) corroborate the inflammatory state at the whole-animal level in every one of the six diseases [6,12,14,18,21,79]. Dry-matter intake at the diagnosis week was reduced in all six (ketosis 14.5, lameness 13.3, milk fever 13.9, retained placenta 15.6, and subclinical mastitis 16.2 vs. 20.3 kg/d in controls; metritis 32.3 vs. 40.5 kg/d as-fed; all *p* ≤ 0.02), and intake depression was already measurable prepartum in retained placenta (−8 wk: 12.5 vs. 16.3 kg/d, *p* = 0.02), milk fever (−4 wk: 11.3 vs. 15.9 kg/d, *p* = 0.02), and subclinical mastitis (−4 wk: 13.0 vs. 15.9 kg/d, *p* = 0.05), consistent with cytokine-driven sickness behavior preceding clinical disease, although lower prepartum intake may also be a pre-existing trait that predisposes to disease (Section 6.7). Milk yield was correspondingly reduced (significant in five of the six diseases). Most tellingly, milk somatic cell count at the diagnosis week was elevated not only in subclinical mastitis (by definition) but also in ketosis (152 vs. 28 × 10^3^ cells/mL, *p* < 0.01), milk fever (118 × 10^3^, *p* < 0.01), lameness (67 × 10^3^, *p* = 0.03), and metritis (92 × 10^3^, *p* = 0.05): subclinical mammary inflammation surfacing across nominally non-mammary diseases, consistent with, though not specific to, a shared syndrome that involves the mammary gland.

Lactate is the most uniformly elevated marker (Figure 6b), and its uniformity is reinforced by the addition of ketosis. It is elevated in 6/6 diseases at −8 wk (four significant; the ketosis cohort shows log2FC = +1.24, *p* = 0.01) and 6/6 at −4 wk (five significant). Both timepoints are well before any clinical sign of disease and remain shifted as disease approaches. Elevated peripheral lactate is the canonical metabolic readout of TLR4-driven aerobic glycolysis: TLR4 → NF-κB → HIF-1α → glycolytic gene upregulation → lactate accumulation in activated macrophages and other immune cells [65,66]. The fact that this signature is universal across all six diseases and present 8 weeks before clinical onset is consistent with the LPS hypothesis, although peripheral lactate can also rise with altered tissue perfusion, catecholamine and glucocorticoid action, or reduced hepatic lactate clearance. The IL-6 signal in ketosis cows is even more striking: log2FC = +3.25 at −8 wk (~10-fold elevation, *p* < 0.01), and the original Zhang et al. 2016 [79] authors themselves speculated that this prepartum cytokine elevation suggested “an endotoxin insult” prior to clinical disease, which is the upstream perturbation hypothesized here.

Cytokines (TNF-α, IL-6) and acute-phase proteins (SAA, haptoglobin) progressively rise as the disease window approaches [81], with TNF-α reaching 6/6 concordance by −4 wk and at disease diagnosis (four significant; ketosis log2FC = +3.03 at Dx). Equally informative is what is not conserved: NEFA and BHBA, the canonical markers of negative energy balance, do not move in a consistent direction across the six diseases at the prepartum timepoints. The shared preclinical state is therefore inflammatory and glycolytic rather than simply energy-deficient—an argument against the view that lipomobilization alone explains the cross-disease convergence.

LBP shows a bimodal pattern that may be biologically informative, although methodological explanations must first be considered (Section 6.3). LBP is hepatically synthesized in response to systemic LPS exposure and serves to chaperone LPS to CD14/TLR4 [32,71,72]; it is a dynamic acute-phase reactant, not a direct quantitative measure of circulating LPS. It is elevated, most clearly during the prepartum window, in three of the five diseases (milk fever, lameness, metritis), consistent with a larger LPS-driven hepatic acute-phase response, and reduced (or unchanged) in two (retained placenta, subclinical mastitis). At first inspection, this pattern might appear inconsistent with a single LPS-driven syndrome. A re-interpretation is offered in Section 6.3: retained placenta is the clinical endpoint of failed placental separation and expulsion associated with a prepartum immune-inflammatory disturbance [18,82], and subclinical mastitis, although carrying the same conserved prepartum signature, does not measurably elevate blood LBP, in contrast to clinical mastitis [83,84]. The bimodality therefore partitions the five evaluable diseases into two empirical response phenotypes—LBP-high (MF, LAM, MET) and LBP-low (RP, SCM)—within the same upstream syndrome; candidate explanations for the LBP-low profile are weighed in Section 6.3. Such response-phenotype structure is difficult to detect in single-disease analyses; whether it is biological or methodological is examined in Section 6.3.

### 4.2. The Urinary Metabolome Shows the Syndrome Strengthening Through the Dry-Off Period and Partially Diverging at Clinical Disease

**Figure 7:**
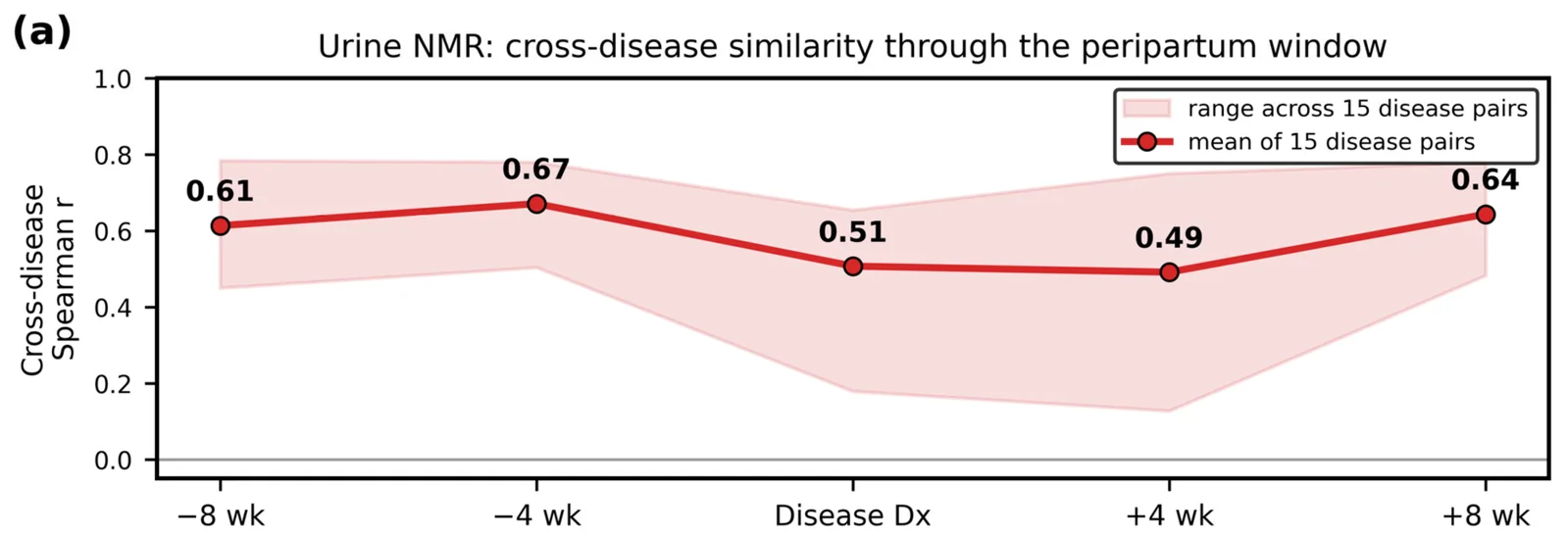

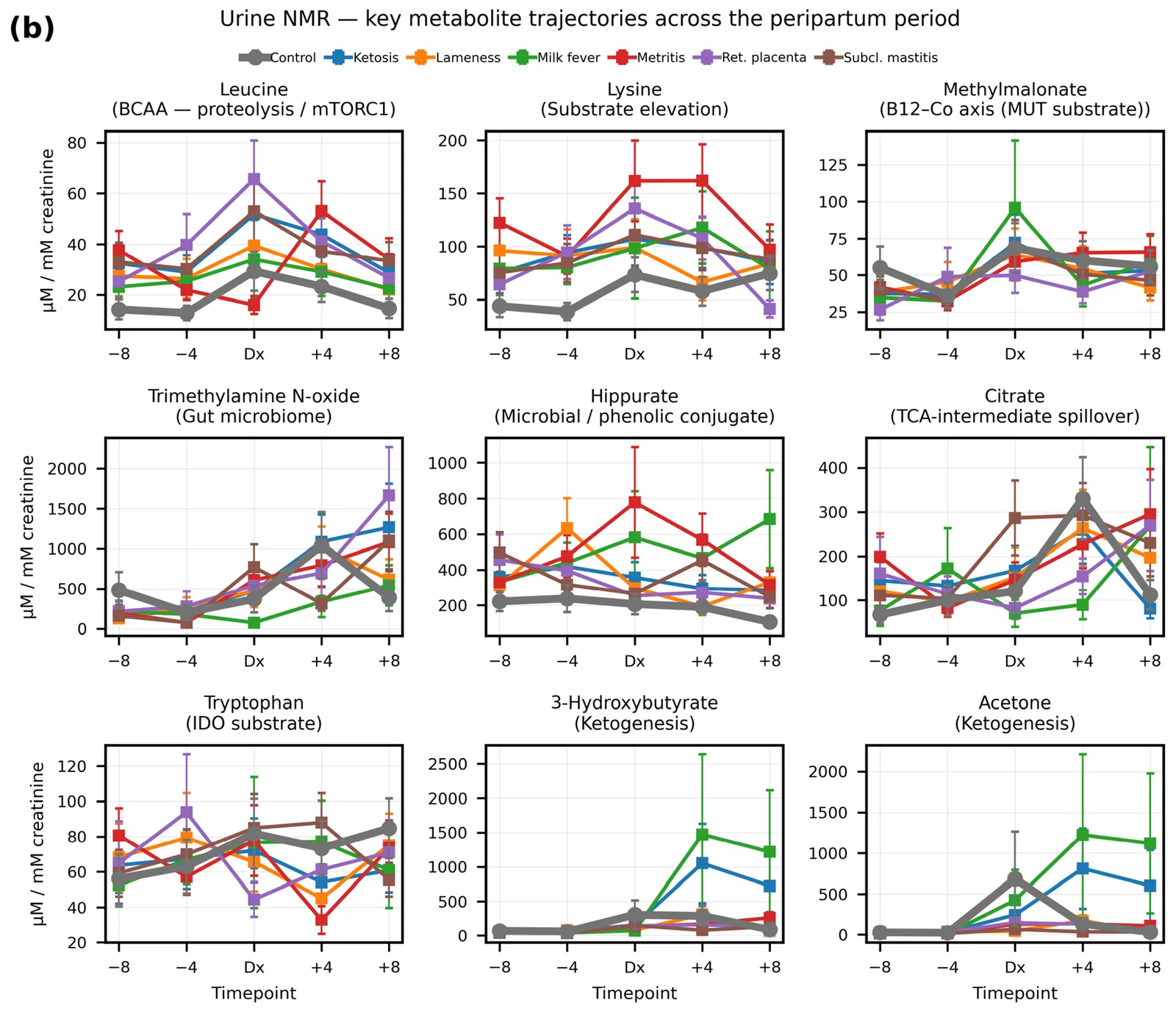
(**a**) Cross-disease similarity in urine NMR through the peripartum window. The mean off-diagonal Spearman r between the six disease log2 fold-change vectors is positive at every timepoint. The signature peaks at −4 wk (r = 0.67), partially diverges at disease diagnosis and +4 wk (r = 0.51 and 0.49, respectively) as individual organ failures manifest and, under the hypothesis, rumen-derived LPS adds to the systemic load, then reconverges at +8 wk (r = 0.64). Reconvergence of direction does not by itself imply resolution; the magnitude of deviation from controls is examined in Section 4.4. The shaded band indicates the range across the fifteen disease pairs at each timepoint. (**b**) Trajectories of nine key urinary metabolites across the peripartum window in each disease group versus control (CTR, thick gray). Leucine and lysine elevation is sustained throughout. Methylmalonate is broadly stable, with disease-specific elevations at disease diagnosis. TMAO shows an inverse pattern: depleted through the dry-off and disease window, then sharply elevated at +4/+8 wk, suggesting a microbiome-host axis reset. Acetone and 3-hydroxybutyrate display a clean disease-specific elevation in ketosis and milk fever at +4/+8 wk, consistent with their role as ketone bodies and markers of increased ketogenesis.

Urine ^1^H-NMR profiles are available for the entire peripartum window (−8, −4, disease, +4, +8 wk relative to calving) for all six diseases plus controls. Disease-specific urinary fingerprints from this cohort have been reported for ketosis [4], lameness [9,10], metritis [17], and subclinical mastitis [23]; here, the six datasets are crossed against one another. Under the cohort-overlap sensitivity analysis introduced in Section 3.2—excluding the comorbid ketosis label—the urinary architecture is unchanged: five-group mean r = 0.57, 0.65, 0.45, 0.45, and 0.61 across the five timepoints (35, 45, 24, 28, and 29 metabolites concordant in all five groups), preserving the −4 wk peak, the dip at diagnosis, and the partial reconvergence at +8 wk. This longitudinal time course allows direct evaluation of Prediction 3 (Section 1.4). The cross-disease Spearman similarity of urinary log2 fold-change vectors is plotted at each timepoint in Figure 7a; the number of urinary metabolites concordant in all six diseases at each timepoint is plotted in Figure 7b.

The temporal structure is consistent with the pattern anticipated by the mammary-origin LPS hypothesis (Section 1.4). Cross-disease coherence is already strong at −8 wk (r = 0.61, with 35 metabolites concordant in 6/6 diseases) and strengthens rather than weakens through the dry-off period, peaking at −4 wk (r = 0.67; 44 metabolites concordant in 6/6), within the dry period and shortly after the major active-involution phase—a timing compatible with a sustained prepartum mammary contribution, though not specific evidence of mammary origin. By disease diagnosis, the coherence has dropped to r = 0.51 (24 metabolites concordant). Two changes coincide here: individual organ failures manifest, and the single dominant prepartum exposure is replaced by several concurrent ones—continuing mammary involvement, rumen-derived LPS from the new high-concentrate diet, and uterine LPS and LTA released into lochia and absorbed across the involuting endometrium (Section 1.3). A shift from one dominant source to several partly independent sources, each with a different ligand mix, microbial ecology, and time course, would be expected to reduce the coherence of the systemic response even while every cow remains inflamed, which is the pattern observed. The lowest cross-disease coherence is at +4 wk (r = 0.49; 28 concordant), the period of maximum heterogeneity in disease resolution. The serum GC-MS layer behaves the same way (Figure 1): prepartum coherence (r = 0.49) drops at diagnosis (r = 0.36), so the divergence-at-disease phase is not a urine-specific phenomenon. By +8 wk, the coherence has partially recovered (r = 0.64; 27 concordant) as the disease groups return toward a common profile. Whether that shared profile represents resolution or a persisting subclinical disturbance cannot be settled by coherence alone; Section 4.4 addresses it directly and finds the latter.

This biphasic architecture (clean rise, peak just before disease, divergence at disease, partial reconvergence thereafter) matches the temporal pattern anticipated for a mammary-origin LPS trigger followed by a more diffuse postpartum amplification (Section 1.3). It is less readily reconciled with a generic parturition-stress framework, which would predict either a sharp peak at parturition followed by recovery, or a uniform plateau. It is also less readily reconciled with a rumen-LPS-only framework, which would anticipate the strongest signal after calving rather than before. Prediction 3 is therefore supported, with the caveat that this time course is also compatible with other drivers that begin before calving, including glucocorticoid-associated reprogramming, pre-existing differences in intake or body condition, and gut-derived signals (Section 6.7).

### 4.3. Specific Urinary Metabolites Reveal Multi-Tissue Mechanistic Coherence

**Figure 8:**
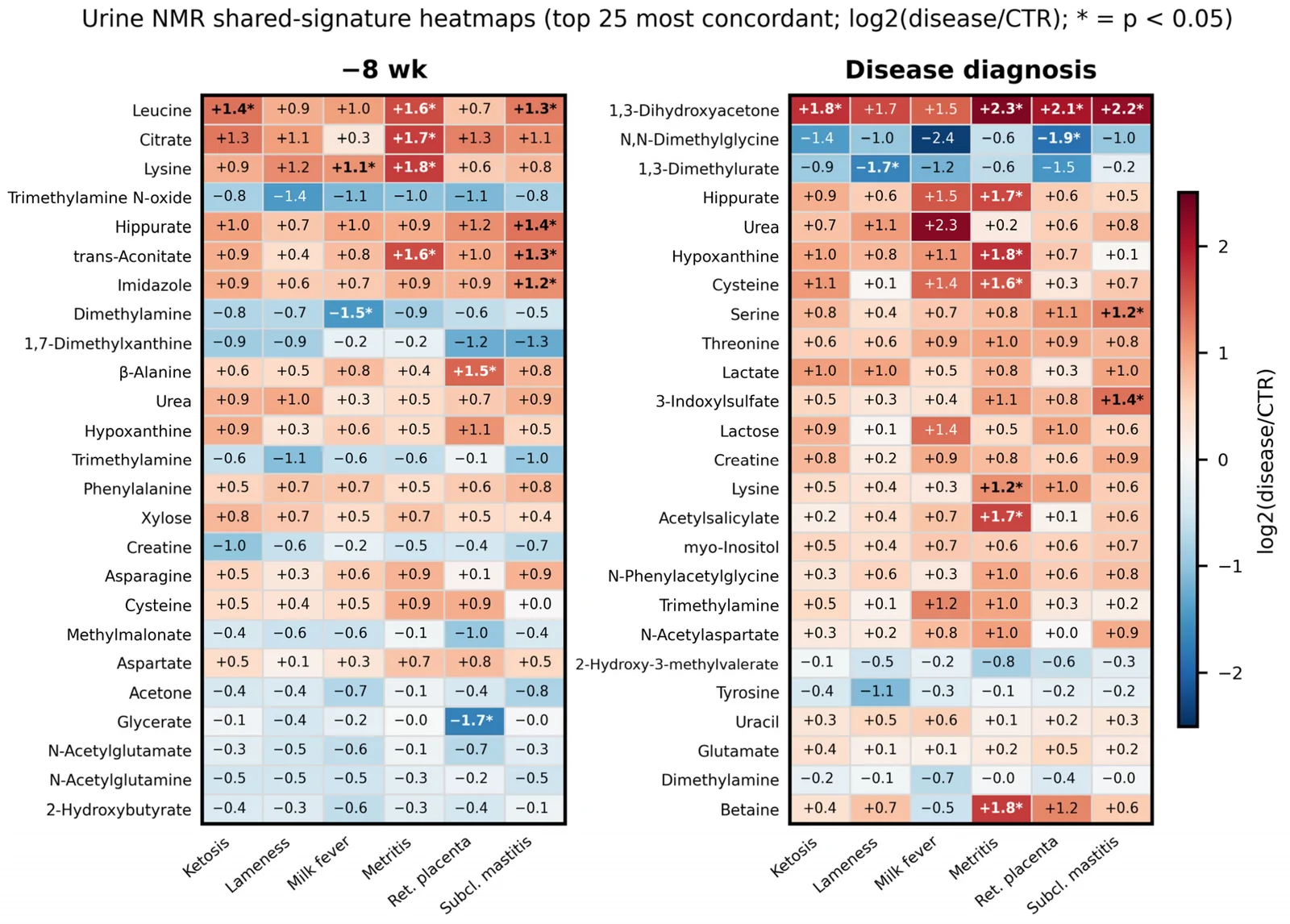
Urine NMR shared-signature heatmaps at −8 wk (**left**) and disease diagnosis (**right**). At −8 wk the conserved signature is centered on branched-chain amino acids, lysine, citrate, hippurate, and trans-aconitate (all elevated), with simultaneous reduction of microbial-derived methylamines (TMAO, TMA, dimethylamine) and methylmalonate. At disease diagnosis, the dominant signal shifts to elevated 1,3-dihydroxyacetone and reduced N,N-dimethylglycine and 1,3-dimethylurate. The evolution of the dominant metabolites between timepoints reflects the syndrome shifting from anticipatory metabolic preparation to active disease metabolism.

The metabolites driving the urinary signature are biologically informative (Figure 8). Three observations stand out.

First, branched-chain amino acids (BCAAs) and lysine are elevated in both serum and urine in all six diseases. This argues against a parsimonious alternative explanation in which serum BCAA elevation reflects reduced renal clearance: if clearance were reduced, urinary BCAAs should be reduced rather than elevated. Both serum and urinary BCAAs being elevated suggests that the host is releasing more, i.e., proteolytic mobilization of muscle protein and reduced amino acid uptake into the immune cells consuming them, both of which are documented downstream of TLR4/mTORC1/cachexia signaling and also of glucocorticoid-driven muscle proteolysis (Section 6.7).

Second, three microbial-derived methylamines are concordantly reduced in 6/6 diseases at −8 wk: trimethylamine N-oxide (TMAO; −1.07 log2FC mean), trimethylamine (TMA; −0.65), and dimethylamine (−0.82). These metabolites are generated by gut microbial conversion of dietary choline, carnitine, and betaine; their concurrent reduction across all six diseases is consistent with altered microbial–host methylamine metabolism in disease-prone cows during the dry-off period. This is consistent with the recent literature on the gut–host axis in transition cows [50] and raises the possibility of a gut contribution acting alongside, or instead of, the mammary source (Section 6.7). The two are not mutually exclusive; the urinary methylamine signature is consistent with shifting microbial–host co-metabolism in disease-prone cows even during the forage-based dry-off period, despite the absence of grain-induced acidosis.

Third, urinary methylmalonate is reduced in all six diseases at −8 wk (−0.50 log2FC mean). Methylmalonate is the substrate of methylmalonyl-CoA mutase, the vitamin B12 (cobalt-dependent) enzyme that converts methylmalonate to succinyl-CoA for entry into the TCA cycle. Reduced urinary methylmalonate is consistent with the substrate being consumed more efficiently by an active enzyme, a hypothesis-generating association rather than a direct flux measurement. This finding sets up the multi-tissue cobalt-B12 coherence story developed in Section 5.2: milk cobalt is elevated, urine methylmalonate is reduced, serum sarcosine is elevated, and all three findings are coherent with peripheral one-carbon and propionate-utilization upregulation.

### 4.4. The Disturbance Persists Subclinically After the Clinical Episode

The analyses above indicate that the shared signature is present eight weeks before calving and remains detectable at clinical diagnosis. That pattern is still compatible with a weaker reading of the hypothesis: that the six diseases share a prepartum risk state which then gives way to six unrelated disorders that resolve once treated. The postpartum samples address a different and practically consequential question: whether the metabolic disturbance ends when the clinical signs do, or continues subclinically after a cow has been recorded as recovered.

**Figure 9:**
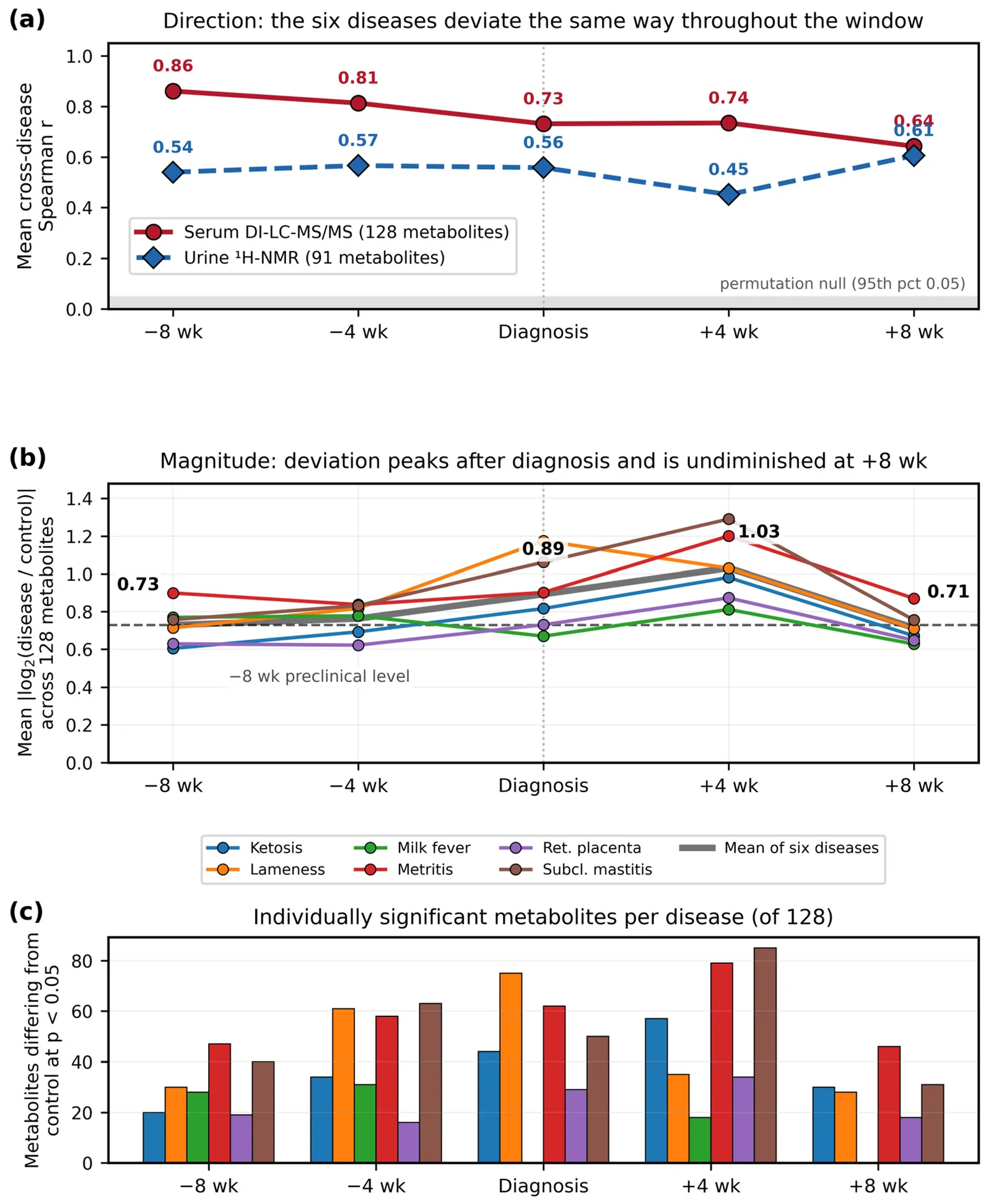
Deviation from controls had not returned to baseline by 8 weeks postpartum. (**a**) Mean pairwise cross-disease Spearman correlation between log2 fold-change vectors at each timepoint, measuring whether the six diseases deviate from control in the same direction; red solid line with circles, serum DI-LC-MS/MS (128 metabolites); blue dashed line with diamonds, urine ^1^H-NMR (91 metabolites). The shaded band marks the 95th percentile of a permutation null generated by shuffling metabolite order independently within each disease vector. (**b**) Mean absolute log2(disease/control) across the 128 serum metabolites for each disease; heavy gray line, mean of the six diseases; dashed line, the −8 wk preclinical level. (**c**) Number of metabolites differing individually from control at *p* < 0.05, per disease. Panel (**b**) values are subject to small-sample upward bias (Section 4.4). Panels (**a**,**b**) are complementary and must be read together: correlation alone cannot distinguish resolution from a disturbance that persists along a shared axis. The dotted vertical line in panels (**a**,**b**) marks the week of diagnosis. In panels (**b**,**c**), colors identify the diseases as shown in the legend (blue, ketosis; orange, lameness; green, milk fever; red, metritis; purple, retained placenta; brown, subclinical mastitis).

The dry-off period could influence not only disease risk but also subsequent lactational and reproductive performance, a possibility that remains to be tested. Two distinct quantities are required, and conflating them would be an error. Cross-disease correlation of log2 fold-change vectors measures whether the diseases deviate from control in the same direction; it carries no information about how far they deviate. Vectors that shrink toward zero as animals recover and vectors that remain large and aligned because a shared disturbance persists can both yield a high correlation. Direction and magnitude are therefore reported together (Figure 9a,b), and only the two together distinguish resolution from subclinical persistence. The effect sizes used in this section are drawn from the complete five-timepoint per-disease tables, which cover all six diseases on both the serum DI-LC-MS/MS (128 metabolites) and urine ^1^H-NMR (91 metabolites) panels; the value they give at −8 wk in serum (r = 0.86) agrees closely with the per-cow estimate of 0.81 reported in Section 3.2; the two were computed independently.

Direction is preserved throughout the window. Mean pairwise cross-disease Spearman correlation in serum was 0.86 at −8 wk, 0.81 at −4 wk, 0.73 in the week of diagnosis, 0.74 at +4 wk, and 0.64 at +8 wk. Every value exceeded a permutation null whose 95th percentile was 0.04, generated by shuffling metabolite order independently within each disease vector so that each disease retained its own distribution of effect sizes while alignment between diseases was destroyed; none of the 500 permutations reached the observed value at any timepoint. Even at its weakest, at +8 wk, the fifteen pairwise values ranged from 0.32 to 0.84, and 99 of 128 metabolites still moved in the same direction in all six diseases.

Magnitude is the more informative quantity for this question, and, on average, it did not decay. Mean absolute log2 fold change across the 128 serum metabolites, averaged over the six diseases, was 0.73 at −8 wk, 0.76 at −4 wk, 0.89 in the week of diagnosis, 1.03 at +4 wk, and 0.71 at +8 wk. Deviation from contemporaneous controls therefore peaked not at clinical diagnosis but four weeks after it, and the value at +8 wk was indistinguishable from the −8 wk value of 0.73, the same preclinical signature that preceded clinical disease by two months. The number of metabolites differing individually from control at *p* < 0.05 averaged 31, 44, 43, 51, and 26 per disease across the five timepoints (Figure 9c). The published urinary studies of this cohort point the same way. In ketosis, 16 and 31 urinary metabolites differed from controls at +4 and +8 wk, respectively [4]; in lameness, 49 urinary metabolites differed at +8 wk, more than at any earlier timepoint [10], and hippurate, pantothenate, and 1,3-dihydroxyacetone were still altered at +8 wk [9]; and in metritis, urinary alterations continued at +4 and +8 wk [17].

Figure 9b resolves this average by disease; the values below are read from the per-disease tables underlying the figure and are given to two decimals. At −8 wk, the mean absolute deviation ranged from approximately 0.60 (ketosis) to 0.90 (metritis). Deviation rose in every disease after calving, but not synchronously: it peaked in the diagnosis week in lameness (≈1.17) and at +4 wk in subclinical mastitis (≈1.29), metritis (≈1.20), ketosis (≈0.98), retained placenta (≈0.87), and milk fever (≈0.81). At +8 wk, values ranged from approximately 0.63 (milk fever) to 0.87 (metritis): ketosis (≈0.66) and retained placenta (≈0.65) remained at or slightly above their own −8 wk levels; lameness, metritis, and subclinical mastitis were close to them; and milk fever was below its −8 wk level (≈0.77). The six-disease mean of 0.71 corresponds to an average deviation of about 1.6-fold (2^0.71^) per metabolite, in either direction. Three qualifications apply. First, a similar mean absolute deviation does not mean that the same metabolites are perturbed to the same extent: fewer metabolites differed individually from controls at +8 wk than at −8 wk (mean 26 versus 31; Figure 9c), so the +8 wk average is carried by a smaller set of larger shifts. Second, the mean absolute log2 fold change is biased upward in small groups, because sampling noise alone produces non-zero absolute values and the bias increases as group size falls; the small case groups (six cows, and four milk fever cows after calving) therefore inflate the magnitudes to an unknown degree, most for milk fever after calving. Third, a high cross-disease correlation together with this magnitude indicates a shared direction of deviation, not an undiminished degree of pathology; neither quantity measures clinical or functional impairment. The +8 wk data are therefore best described as showing that deviation from controls had not returned to baseline, rather than that the pathological perturbation was unchanged.

Taken together, these two quantities argue against complete metabolic resolution by +8 wk. At eight weeks postpartum the six disease groups still deviated from their contemporaneous controls by as much as they had eight weeks before calving, and still deviated in a direction shared across all six diagnoses. The reconvergence of coherence after +4 wk is therefore more consistent with the return of a common disturbance than with recovery, after the transient heterogeneity introduced by six different organ failures and by the shift from one dominant prepartum LPS source to several concurrent postpartum ones (Section 4.2). The clinical episode ends, whereas the metabolic deviation appears to persist.

Five limits should be stated plainly. The milk fever group was reduced to four cows after calving, so its estimates at +4 and +8 wk are less precise. Clinical recovery was not independently scored at +8 wk, so persistence is characterized metabolically rather than clinically. Significance counts for milk fever at the diagnosis week and at +8 wk are conservative, because no individual metabolite reached *p* < 0.05 at those timepoints in the source tables; the direction and magnitude estimates, which do not depend on per-metabolite significance, are unaffected. The magnitude estimates are subject to the small-sample bias described above. And the design is observational throughout: the trajectory is consistent with a common endotoxin-driven program but does not by itself establish that LPS is the cause.

## 5. The Mammary Gland Appears Metabolically Engaged

### 5.1. The Milk Metabolome at +2 wk Shows the Same TLR4-Pattern Shift

**Figure 10:**
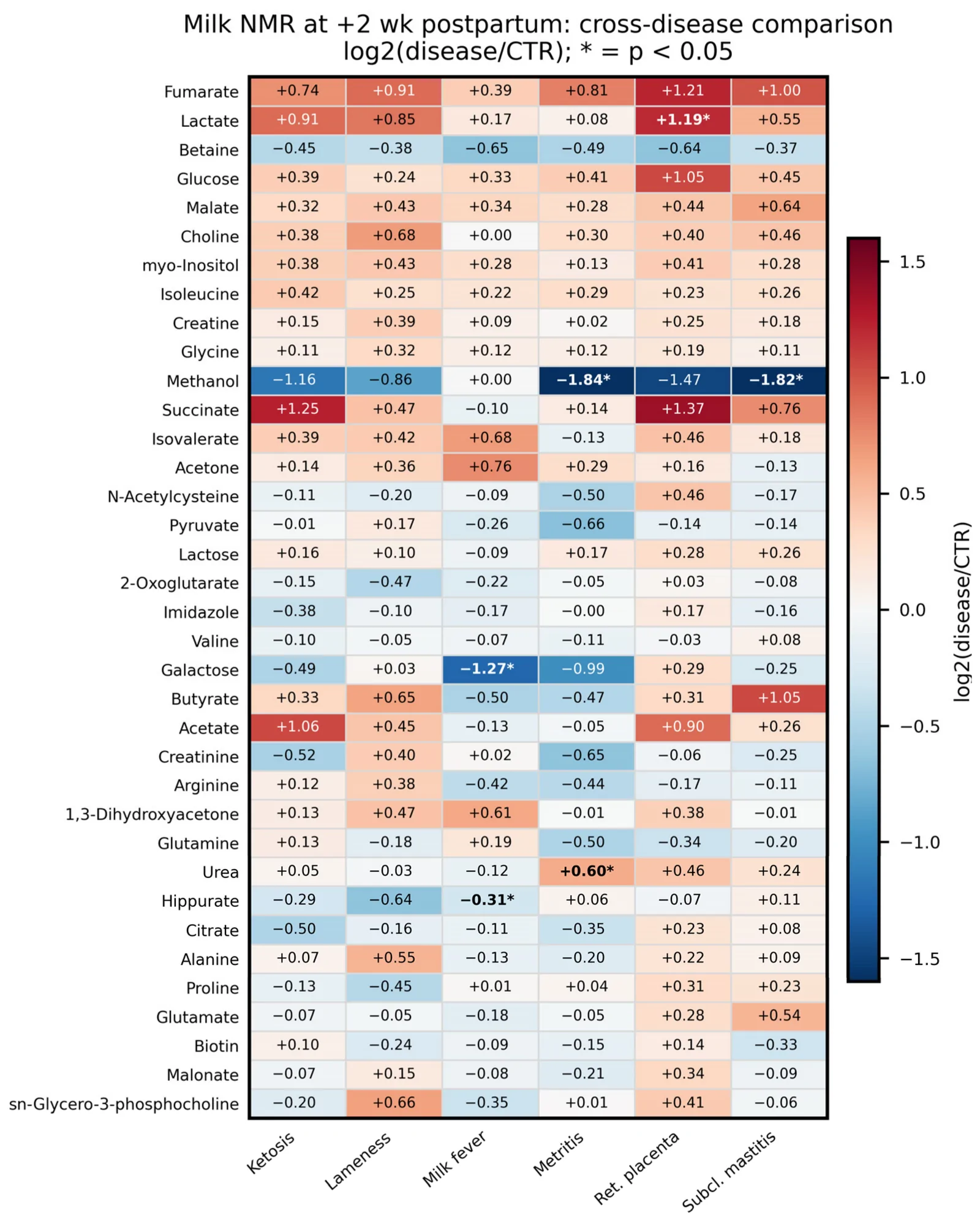
Milk NMR cross-disease comparison at +2 wk postpartum. Color encodes log2(disease/control); asterisks denote *p* < 0.05. Mean cross-disease Spearman’s r = 0.52. Ten metabolites are concordantly altered in all six diseases: nine elevated (fumarate, lactate, glucose, malate, choline, myo-inositol, isoleucine, creatine, glycine) and one reduced (betaine). The pattern reveals three coherent biological themes: (i) TCA-cycle intermediate spillover (fumarate, malate, succinate); (ii) methylation flux disruption (choline ↑, betaine ↓); (iii) aerobic glycolysis shift (lactate ↑, glucose ↑)—all hallmarks of TLR4-driven cellular metabolic reprogramming [65,66].

Of all the data layers, the milk metabolome carries the highest mechanistic specificity for the LPS hypothesis, because milk is the secretion of the mammary gland, the candidate source organ of the syndrome trigger. Milk metabotyping in this cohort has already shown disease-specific alterations, for example in lameness [11], and blood metabotyping of dry cows predicts elevated early-lactation milk somatic cell counts [25], both consistent with mammary involvement in the broader syndrome. If the mammary gland were merely on the receiving end of systemic inflammation, milk should reflect generic plasma-equilibration changes; if the mammary gland is itself metabolically engaged in the syndrome, milk should display the same TLR4-pattern signature as serum and urine but expressed locally by mammary epithelium. The data are more consistent with the latter (Figure 10).

Three biological themes are evident in the milk signature, each consistent with active TLR4 engagement of the mammary epithelium itself. First, the TCA-cycle intermediates fumarate and malate, together with lactate, are concordantly elevated in milk in 6/6 diseases, and succinate in 5/6 (all but milk fever). TCA-intermediate spillover into the secreted milk pool is a hallmark of mitochondrial dysregulation in inflamed cells, and pyruvate-to-lactate conversion with lactate accumulation is the canonical readout of HIF-1α-driven aerobic glycolysis [65]. The succinate finding is of particular interest: succinate accumulation in TLR4-activated macrophages is the proximate signal that stabilizes HIF-1α and drives IL-1β transcription, as established by Tannahill et al. [65]. Finding succinate elevated in milk across five of the six peripartum diseases suggests that the mammary epithelium is exhibiting the same metabolic reprogramming.

Second, choline is elevated and betaine is reduced in milk in 6/6 diseases, a signature of active betaine→glycine remethylation, in which betaine donates a methyl group to homocysteine to regenerate methionine, with consequent betaine depletion. Glycine is also elevated in 6/6, consistent with this pathway. The methylation-flux disruption is internally coherent with the elevation of serum sarcosine (a glycine N-methyltransferase product; Section 3.3) and provides a tissue-localized echo of the same one-carbon flux disruption visible systemically.

Third, lactose, glucose, and lactate are all elevated in milk in 5/6 or 6/6 diseases. Glucose elevation in milk reflects altered mammary glucose uptake; lactate elevation reflects either mammary aerobic glycolysis or local immune-cell glycolysis within mammary tissue (during subclinical mastitis particularly, infiltrating neutrophils contribute to milk lactate). The combination of glucose and lactate elevation is the metabolic shift expected if the mammary epithelium itself is running TLR4-driven aerobic glycolysis. This is the strongest single observation in favor of active mammary engagement, although milk glucose and lactate also vary with infiltrating leukocytes, milk yield, and blood–milk barrier permeability, and postpartum samples cannot show whether the gland was a source of LPS during the dry period. A deeper 124-metabolite DI-LC-MS/MS milk panel from the same cohort adds a complementary, and boundary-setting, layer. Cross-disease similarity remains positive on average (mean r = 0.35, the weakest of the five metabolomic layers, with one disease pair, lameness–metritis, near zero), and seventeen metabolites are concordantly altered in all six diseases, roughly four times the chance expectation, with the concordant set dominated by phosphatidylcholine species elevated in milk (PC aa C30:0 and PC aa C32:1 significant in 5/6 diseases): the membrane-phospholipid remodeling theme documented in serum (Section 3.3) reappears in the secretion of the source organ itself. Notably, milk kynurenine is not concordantly elevated, indicating that the IDO1 axis is a systemic (serum) signature rather than a milk-secreted one, consistent with the milk profile reflecting local epithelial metabolic reprogramming rather than passive equilibration with plasma.

### 5.2. Milk Minerals Reveal Coherent Multi-Tissue B12-Cobalt Axis Activation

**Figure 11:**
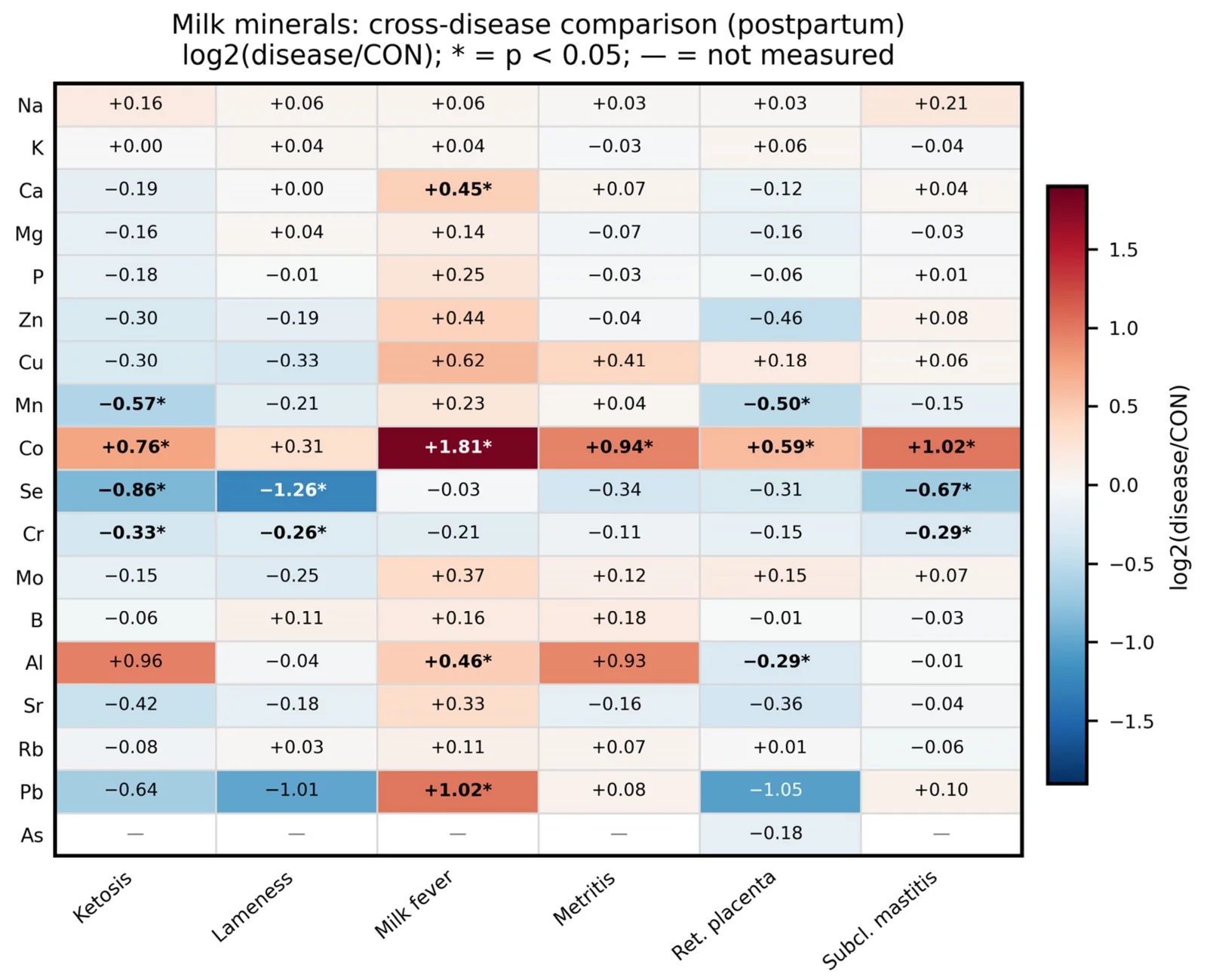
Milk minerals across the six peripartum diseases (postpartum). Color encodes log2(disease/control); asterisks denote *p* < 0.05. Cobalt is elevated in 6/6 diseases (5/6 significant, with a non-significant increase in lameness). Selenium and chromium are reduced in 6/6 diseases (3/6 significant for each). The cobalt finding connects to the urinary methylmalonate and serum sarcosine results to form a coherent multi-tissue picture of B12-dependent peripheral metabolism upregulated under sustained low-grade inflammation.

Milk minerals were measured by ICP-MS for 18 elements across all six diseases (arsenic only in retained placenta; Figure 11). A precedent exists within this cohort: serum metallotyping of ketotic cows identified mineral alterations preceding, accompanying, and following the disease [3]. Three elements are concordantly altered in all six diseases: cobalt is elevated (mean log2FC = +0.91, significant in 5/6 diseases with *p* ≤ 0.04 and a non-significant increase in lameness at *p* = 0.26), selenium is reduced (−0.58, significant in 3/6), and chromium is reduced (−0.23, significant in 3/6).

The cobalt elevation is biologically the most diagnostic of the mineral findings. Cobalt is the metal cofactor of vitamin B12 (cobalamin), which serves as the cofactor for two bovine enzymes of central peripheral metabolism: methionine synthase (one-carbon flux, methylation reactions) and methylmalonyl-CoA mutase (propionate utilization, the bovine entry point for ruminal short-chain fatty acids into the TCA cycle). Elevated cobalt in milk, with effect sizes ranging up to log2FC = +1.81 in milk fever (*p* = 0.0001), means the cow is exporting more cobalt to milk, either because B12 turnover is upregulated and more cobalt is being moved through the pathway, or because the inflamed mammary epithelium handles cobalt differently. Both interpretations are consistent with the LPS-driven syndrome model.

Critically, the cobalt finding is internally coherent with two independent findings in two other tissues. In urine, methylmalonate (the substrate of the cobalt-B12-dependent enzyme methylmalonyl-CoA mutase) is reduced in 6/6 diseases (Section 4.3); reduced urinary substrate is consistent with increased enzymatic consumption, i.e., active B12-dependent flux. In serum, sarcosine (a marker of methionine-cycle one-carbon flux, also B12-dependent through methionine synthase) is elevated in 6/6 diseases (Section 3.3). Three findings, in three different tissues, on three different analytical platforms, are all compatible with the same biological pathway. Such multi-tissue coherence cannot be observed in single-disease studies and is the type of signal a syndrome-level analysis is designed to detect. However, the three measurements were not made at the same time (serum and urine prepartum, milk postpartum), and none directly measures vitamin B12 status or enzyme flux, so the B12 interpretation remains a hypothesis.

The selenium reduction is consistent with selenoprotein consumption during oxidative burst. Selenium is the catalytic atom of glutathione peroxidase and several other selenoproteins; activated neutrophils and macrophages consume selenium via glutathione peroxidase 1 to neutralize the hydrogen peroxide generated during the respiratory burst. Reduced milk selenium across 6/6 diseases is consistent with peripheral selenium being preferentially consumed by activated immune cells rather than partitioned into milk, a tissue-level redistribution that would be expected under sustained low-grade systemic inflammation.

## 6. Discussion—A Proposed Unifying Mechanism: LPS-Driven Metabolic-Immune Syndrome

### 6.1. Evaluation of the Four Predictions

The four predictions of the single-syndrome hypothesis enumerated in Section 1.4 are evaluated against the data of Section 3, Section 4 and Section 5 as follows. Here, “supported” means that the observations agree with the prediction and do not refute it; it does not mean that the prediction has been confirmed causally (Section 2.7).

Prediction 1 (cross-disease similarity uniformly positive): supported. All eleven cross-disease similarity measurements across the five metabolomic data layers are positive at the mean level, exceed a shared-control-aware permutation null, and survive cross-fitted and bootstrap sensitivity analyses (Section 3.1), with mean r = 0.35–0.81 (Figure 1; Section 3.1).

Prediction 2 (a conserved molecular signature mapping onto TLR4/IDO/HIF-1α/mTORC1/PLA_2_ downstream pathways): supported. Thirteen serum metabolites concordant in 6/6 diseases are compatible with these pathways (Figure 3 and Figure 4; Section 3.3 and Section 3.4). The Kyn/Trp ratio (the canonical IDO1 readout) is elevated 3–4× in every disease at *p* = 8.7 × 10^−6^. Lactate (the canonical HIF-1α-glycolysis readout) is elevated in 6/6 diseases at −8 and −4 wk and in milk in 6/6 diseases.

Prediction 3 (biphasic temporal architecture: clean mammary signal prepartum, mixed signal postpartum): supported, with qualifications. Urinary cross-disease coherence rises from r = 0.61 at −8 wk to peak at r = 0.67 at −4 wk (proposed mammary-dominant window), drops to r = 0.49–0.51 at disease and +4 wk (postpartum multi-source window), and partially reconverges at r = 0.64 at +8 wk (Figure 7a; Section 4.2). The reconvergence is more consistent with a persisting disturbance than with recovery: mean deviation from contemporaneous controls at +8 wk was similar to the −8 wk preclinical level in serum and was maximal at +8 wk in urine (Figure 9; Section 4.4). The same time course is, however, also compatible with non-LPS prepartum drivers (Section 6.7).

Prediction 4 (convergent evidence across biofluids, with milk providing source-organ-informative evidence): supported by cross-matrix convergence and postpartum mammary engagement, although the largest cross-disease correlation was observed in serum. The milk metabolome shows the same TLR4-pattern shift as serum and urine, and the milk minerals reveal a coherent multi-tissue B12-cobalt axis (Figure 10 and Figure 11; Section 5). The mammary gland is implicated as actively metabolically engaged postpartum, not merely a downstream tissue. Overall, the four predictions receive varying degrees of support in this exploratory integrative re-analysis; the findings are consistent with, but do not by themselves establish, a shared LPS-associated metabolic-immune syndrome or its proposed mammary origin.

### 6.2. The LPS Hypothesis as a Parsimonious, but Not Exclusive, Explanation

Several alternative frameworks could in principle explain pieces of the data, and the LPS hypothesis appears to account for the largest share of the dataset within a single framework. Energy-deficit/negative-energy-balance frameworks explain ketosis-related findings (acetone and 3-hydroxybutyrate elevation in ketosis-prone cows at +4/+8 wk; Figure 7b) and partial BCAA mobilization, but they do not predict cross-disease similarity in non-energy-deficit diseases (lameness, retained placenta, subclinical mastitis), nor the Kyn/Trp axis activation, nor the lipid-mediator signature, nor the milk-mineral findings. Calcium-deficit frameworks explain milk fever specifically but not the other five diseases, which is the wrong scope for explaining cross-disease coherence. Generic parturition-stress frameworks do not readily explain why the cross-disease coherence is highest at −4 wk (well before parturition) rather than at parturition itself, and they do not predict the specific TLR4-pattern signature observed. Other candidates—namely glucocorticoid-driven metabolic reprogramming, pre-existing nutritional differences, and a primary gut-microbial driver—are not excluded by these data and are evaluated in Section 6.7.

In contrast, the LPS hypothesis anticipates most components of the observed signature: TLR4 → NF-κB → cytokines (TNF-α, IL-6 elevation, observed); HIF-1α → aerobic glycolysis (lactate elevation, observed in serum, urine, and milk; succinate elevation in milk, observed); IDO1 → Kyn axis (2.8–4.0× elevation, observed in every disease); mTORC1 → BCAA mobilization (Leu, Ile, Lys elevation, observed in serum and urine); PLA_2_ → lysoPC release and SM remodeling (lysoPC a C18:0, PC aa C30:2, three SM(OH) species elevation, observed); hepatic acute-phase response (SAA and haptoglobin elevated across diseases; LBP elevated in the LBP-high phenotype, Section 6.3); peripheral B12/one-carbon flux upregulation (cobalt ↑ in milk, methylmalonate ↓ in urine, sarcosine ↑ in serum, all observed); selenium consumption by oxidative burst (selenium ↓ in milk, observed); microbiome-host axis disturbance (TMAO, TMA, dimethylamine ↓ in urine, observed). The data are therefore consistent with the multi-tissue signature expected of low-grade endotoxemia—a mammary-dominant prepartum phase giving way to mixed mammary, ruminal, and uterine exposure after calving, with the ligand pool broadening from LPS alone to LPS plus Gram-positive LTA and the coherence of the systemic response falling accordingly—in line with the foundational framework laid out by Eckel and Ametaj [32] and the broader metaflammation literature [74,75]. None of these components is, however, specific to LPS; each can also be produced by other inflammatory, endocrine, or nutritional stimuli.

### 6.3. The LBP Bimodality May Identify Two Response Phenotypes Within the Syndrome

The bimodality of LBP across the five evaluable diseases (Figure 5; Section 4.1) deserves a careful re-interpretation. LBP is hepatically synthesized in response to systemic LPS exposure and inflammatory cytokines, particularly IL-6, and serves to chaperone LPS to CD14/TLR4 [32,71,72]; elevation in milk fever, lameness, and metritis is consistent, with these cows mounting the largest hepatic acute-phase response to systemic LPS exposure. Because LBP is a dynamic hepatic reactant rather than a direct quantitative measure of circulating LPS, the contrasting pattern is best read as identifying empirical LBP-high and LBP-low response phenotypes, whose candidate mechanisms are weighed below rather than asserted. In retained placenta, reduced LBP is consistent with a disease that is not itself a primary systemic LPS event but the clinical endpoint of failed placental separation and expulsion associated with a prepartum immune-inflammatory disturbance: impaired neutrophil function precedes RP [82], a systemic inflammatory state is already present at −8 and −4 wk in cows that later develop RP [18], and the subsequent LPS exposure is more compartmentalized (intrauterine) than in primary metritis. This framing respects the established epidemiological sequence in which RP precedes and predisposes to metritis [28]: the claim is not that RP follows clinical metritis, but that both share an upstream inflammatory antecedent, which RP expresses first as a failure of immune-mediated placental detachment. Serum LBP has been reported to be elevated in cows with established RP after calving, albeit in a small cross-sectional cohort (nine RP versus six control cows) [85]; the reduced values documented here derive from the prediagnostic window. Sampling time is therefore a plausible, though not demonstrated, reconciliation: the combined evidence suggests a dynamic pattern of reduced or dysregulated LBP before RP, followed by an acute-phase rise once placental retention and postpartum inflammation are established, a pattern that requires longitudinal confirmation in the same cows.

In subclinical mastitis, the LBP-low profile should not be read as evidence that mammary inflammation is physiologically compartmentalized: a stimulus that originates in the mammary gland can nonetheless act systemically. Experimental intramammary LPS challenge alters circulating inflammatory and metabolic indices, hepatic acute-phase gene expression, and liver lipid metabolism [43], demonstrating that a mammary-origin inflammatory stimulus can generate systemic and hepatic consequences, although whether that hepatic response is mediated by translocation of intact LPS, by mammary-derived cytokines, or by both has not been established, and an acute 200 µg bolus is not equivalent to naturally occurring, chronic subclinical infection. What the empirical record shows is that naturally occurring subclinical intramammary infection does not measurably elevate blood LBP, in contrast to clinical mastitis [83], and that, in chronic subclinical mastitis, the acute-phase response is concentrated in the milk of the affected quarter, with serum responses attenuated rather than demonstrably absent [84,86]. Chronic subclinical mastitis may thus represent a predominantly mammary-localized infection with an attenuated, variable, or temporally restricted systemic acute-phase response. The muted LBP could reflect several non-exclusive variables: exposure magnitude, duration, and anatomical site of exposure (in goats, a low-dose intrateat LPS infusion elicits only mild, spatially restricted mammary inflammation [elevated somatic cell count and milk amyloid A without broader marker changes], and the infusion site governs how widely LPS distributes within the gland [87]), sampling time relative to infection, pathogen biology (subclinical infections are etiologically heterogeneous; many are Gram-positive and signal through non-LPS pathogen-associated molecular patterns, though Gram-negative cases occur [39]), hepatic responsiveness, endotoxin tolerance after sustained prior exposure (for which preliminary ex vivo PBMC evidence exists in transition cows [73], and for which in vivo cross-organ evidence exists in goats, where repeated intrauterine LPS exposure attenuates the mammary somatic-cell and cytokine response to a subsequent intramammary challenge [47] and mammary inflammation recedes despite continued exposure [48]; notably, plasma LBP remained elevated in that model even as mammary indices recovered [48], so the demonstrated tolerance dampens local responsiveness rather than hepatic LBP output, a dissociation that itself argues against reading LBP as a direct exposure gauge. This tolerance is, moreover, selective reprogramming rather than generalized immunosuppression: mammary leukocytes from preconditioned goats produce less IL-1β and TNF-α after intramammary LPS while phagocytic activity and CD11b expression are preserved or enhanced [88], so muted cytokine, somatic-cell, or LBP readouts cannot automatically be equated with low exposure or immune failure), nutritional status, and individual variation. None of these possibilities conflicts with the mammary gland’s proposed role as the principal initiating LPS source at dry-off—the involuting gland presents a sustained, low-grade, Gram-negative-dominated translocation surface [32,39]—because the phenotype labels describe the hepatic response, not the anatomical limits of the initiating stimulus. The goat and SARA-challenge experiments described in Section 1.3 reinforce this reading: circulating LBP, local inflammatory intensity, and actual LPS exposure can become dissociated through differences in dose, duration, microbial source, sampling time, nutritional state, barrier integrity, and exposure-induced immune reprogramming. Local infection is thus retained here as an anatomical description, not as a physiological conclusion of containment.

The bimodality may therefore reflect two empirical response phenotypes within the same upstream syndrome: LBP-high (MF, LAM, MET), in which the upstream signal is accompanied by an overt hepatic acute-phase and peripheral inflammatory response consistent with a larger LPS-driven hepatic acute-phase response; and LBP-low (RP, SCM), in which the same conserved metabolic-immune signature is present but the hepatic LBP output is muted, plausibly because the disease is expressed locally (SCM) or is the clinical endpoint of failed placental separation downstream of an earlier inflammatory disturbance (RP), although the alternative explanations enumerated above remain open. These labels are deliberately descriptive rather than mechanistic; converting them into causal subtypes will require direct LPS quantification and paired mammary-hepatic sampling (Section 1.5). The phenotype distinction was only visible because the present analysis crossed multiple diseases against one another; it would have been invisible in any single-disease study, where LBP would have appeared simply as “elevated” or “reduced” without context.

Before these phenotypes are accepted as biological, four methodological explanations must be considered, and none can be excluded with the present data. (i) Sampling time: LBP is an acute-phase reactant whose concentration changes within days, whereas the three sampling points (−8 wk, −4 wk, and the diagnosis week) are four weeks apart; a transient rise between samples, or a rise after the diagnosis week, as reported for established retained placenta [85], would be missed. The diagnosis-week sample also fell at different intervals after calving in different diseases (Section 2.4), so LBP was sampled at different phases of the acute-phase response. (ii) Disease definition: retained placenta (fetal membranes retained beyond 24 h) and subclinical mastitis (a somatic cell count threshold without clinical signs) are defined by criteria that capture milder or more localized states than severe lameness or clinical metritis, so lower systemic LBP may partly reflect case severity rather than a distinct response type. (iii) Sample size: with six cases and six matched controls per disease, statistical power to detect moderate LBP differences is low, the between-cow variance of acute-phase proteins is high, and the distinction between “reduced” and “unchanged” is not statistically robust; the direction of the retained placenta and subclinical mastitis contrasts should therefore be interpreted with caution. (iv) Assay: LBP was quantified with a commercial immunoassay in the source studies [6,12,14,18,21]; values close to the lower limit of quantification, or between-plate variation, would compress group differences, and bovine-specific reference intervals are limited.

The LBP-low profile also poses a genuine challenge to an LPS-centric hypothesis. If LPS were the common trigger in all six diseases, a systemic LBP response would be expected in all of them. Its absence in two diseases means that either the exposure in those cows was too small, too localized, or too intermittent to raise hepatic LBP output; or tolerance-like reprogramming attenuated the hepatic response; or the shared metabolic signature in those cows is driven, at least in part, by non-LPS stimuli such as Gram-positive ligands, glucocorticoids, or tissue injury. The present data cannot distinguish among these possibilities. Serial LBP sampling at shorter intervals, together with direct LPS quantification (Section 1.5) and measurement of soluble CD14 and anti-LPS antibodies, is required to decide whether the LBP bimodality is a biological phenotype, a methodological artifact, or evidence against a purely LPS-driven mechanism.

### 6.4. Multi-Tissue Mechanistic Coherence as Supporting Evidence for a Shared Upstream Trigger

A pattern that is internally coherent across multiple tissues and platforms is harder to explain by chance than any single-tissue signal. The B12-cobalt-methylmalonate-sarcosine signal across milk, urine, and serum (Section 5.2) is one such pattern. The lactate elevation across serum, urine, and milk is another: peripheral lactate accumulation, urinary lactate spillover (where measurable), and milk lactate elevation are all expected if TLR4-driven aerobic glycolysis is operating across multiple tissues simultaneously, and that is what is observed. The BCAA elevation across serum and urine is a third: if BCAA elevation in serum reflected reduced renal clearance, urinary BCAAs should fall; their concordant elevation in both matrices suggests that the host is releasing more BCAAs (proteolytic mobilization) and consuming them less efficiently (reduced peripheral uptake by tissues bypassed in favor of immune-cell mTORC1 demand), the signature of cachexia/immune-priority substrate redirection.

The probability that three coherent multi-tissue signals—B12-pathway, lactate-glycolysis, and BCAA-mTORC1—would all arise across the same six diseases by chance appears low, although these signals are not statistically independent. The likelihood that they would all map onto a single, well-characterized inflammatory signaling axis (TLR4) by chance is also low. The pattern is consistent with a single upstream perturbation acting through a stereotyped downstream program, and one parsimonious candidate for that perturbation, given the dry-off-period mammary involution biology and the lack of documented large alternative LPS sources on forage-based dry-cow rations, is mammary-derived endotoxin; the alternatives considered in Section 6.7 nevertheless remain viable.

### 6.5. Cross-Species Inference and the Broader Metaflammation Framework

The signature documented here in dairy cows—BCAA elevation, IDO/Kyn axis activation, lactate elevation, lysoPC and sphingomyelin remodeling, hepatic acute-phase response, microbiome–host axis disturbance—overlaps componentwise with signatures reported in human postpartum disorders [89,90], peri-operative inflammation, sepsis-tolerance states, type 2 diabetes-associated metaflammation, and obesity-driven low-grade chronic inflammation [74,75]. The dairy peripartum syndrome may therefore be one ecologically extreme example of a much more general phenomenon: sustained low-grade endotoxemia may produce this signature, and the clinical “disease” depends on which organ fails first. The dairy peripartum window—a stereotyped, naturally occurring, large-cohort experiment in low-grade endotoxemia—is an unusually clean system in which to study this general phenomenon, and the cross-disease analytical framework demonstrated here may be portable to analogous human cohorts where multiple comorbid postpartum or postoperative conditions present a similar puzzle of overlapping risk factors.

**Figure 12:**
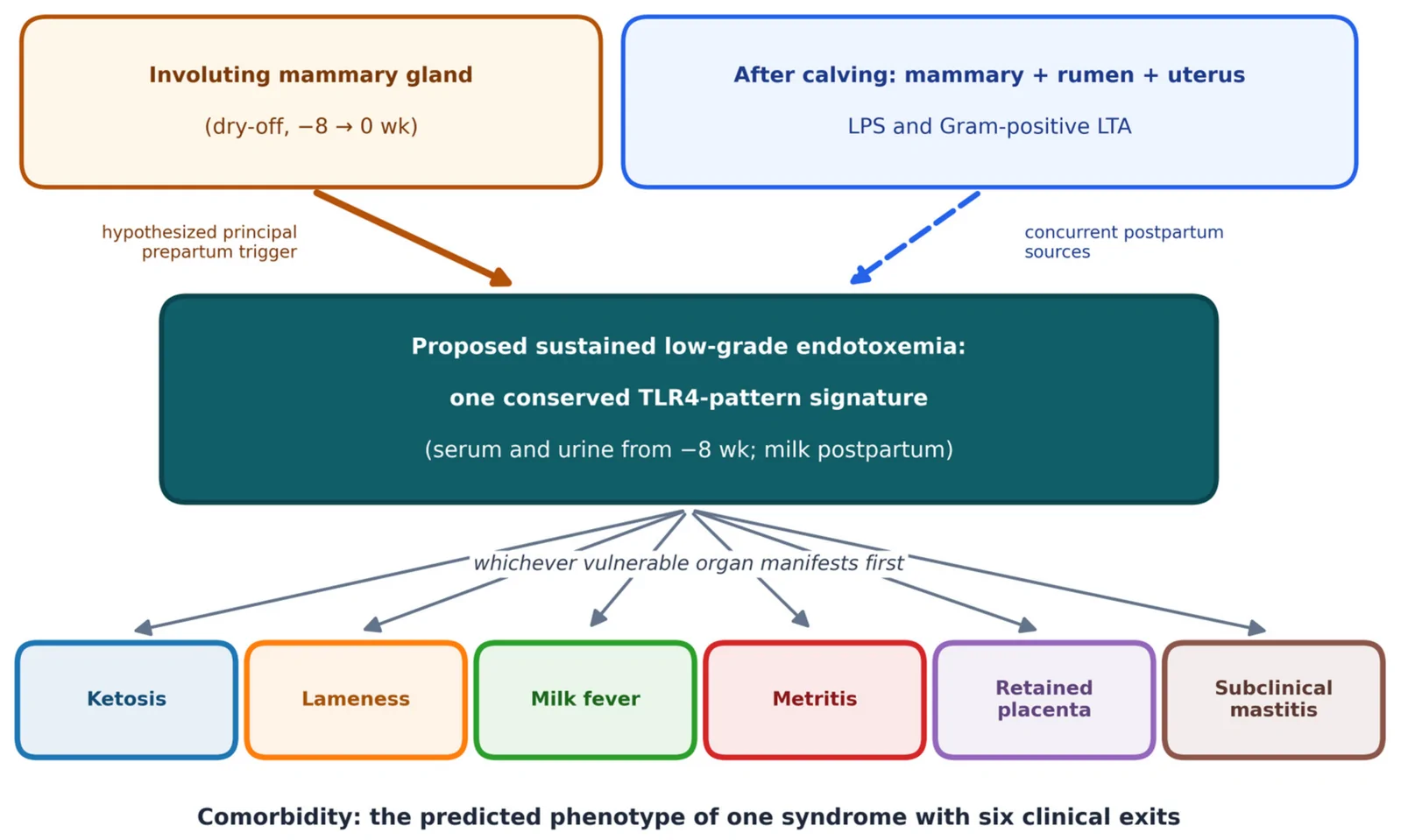
Proposed single-syndrome model. During the dry-off period, mammary involution is hypothesized to provide the principal prepartum source of LPS-associated signaling (solid brown arrow). After calving, three sources act concurrently (dashed blue arrow): continuing mammary involvement and new intramammary infection, ruminal LPS translocation under concentrate-rich feeding, and uterine LPS and Gram-positive lipoteichoic acid released into lochia and absorbed across the involuting endometrium. The resulting conserved TLR4-pattern metabolic-immune signature is detectable in serum and urine from −8 wk pre-calving and in milk postpartum. Clinical expression depends on the first vulnerable organ system to lose function; comorbidity is therefore predicted as multiple clinical exits from a shared upstream syndrome. Direct measurement of circulating and mammary LPS-associated material remains necessary to confirm the proposed source and mechanism.

The mammary-origin LPS hypothesis is therefore not only a hypothesis about dairy cattle. It is a hypothesis about how a stereotyped barrier-disruption event (mammary involution at dry-off) generates sustained low-grade endotoxemia, which then drives a stereotyped TLR4-pattern host response, which then expresses clinically through whichever organ is most vulnerable in any given individual. If the framework is correct, it predicts that intervention strategies that target the upstream barrier-disruption event (e.g., gradual rather than abrupt cessation of milking; prophylactic anti-inflammatory or LPS-binding intervention during dry-off; mammary-microbiome-modulating approaches) should reduce the cumulative incidence of all six diseases simultaneously rather than any one disease individually, a prediction that is testable and that could, if confirmed, shift peripartum dairy management from a disease-specific to a syndrome-prevention paradigm. The proposed single-syndrome model is summarized in Figure 12. The framework also reframes the predictive-biomarker results of the contributing single-disease studies. In every one of the six diseases, the published serum DI-LC-MS/MS biomarker panels drawn from this cohort discriminated future disease from health at −8 and −4 wk, with reported area under the curve (AUC) ≥ 0.99 [2,8,13,15,19,24], performance that certainly requires external validation at these sample sizes, but whose uniformity across six nominally distinct conditions is itself the syndrome prediction: all six panels are sampling one shared upstream signature. Consistent with this, the published significant-metabolite lists overlap heavily through the prepartum window: kynurenine and the branched-chain and substrate amino acids leucine, isoleucine, and lysine are each significantly altered in five of the six diseases at −8 wk, −4 wk, and the diagnosis week, and acylcarnitine C10 in all six at −8 wk [2,8,13,15,19,24]. Cross-herd external validation of a single parsimonious panel, anchored on the Kyn/Trp ratio, is therefore the practical translational next step.

### 6.6. Proposed Mechanistic Sequence from Dry-Off to Clinical Disease

The proposed mechanism can be summarized as a sequence of six steps (Figure 12). Step 3 and parts of steps 2, 5, and 6 are supported by the present data; steps 1 and 4, and the causal links between steps, are hypothetical and require direct testing.

**Step 1: mammary exposure at dry-off.** Abrupt cessation of milking raises intramammary pressure, strains the epithelial barrier, and recruits leukocytes into the involuting gland [37,38], which is also susceptible to new intramammary infection [40,41,42]. Bacterial ligands (LPS from Gram-negative organisms; lipoteichoic acid and other ligands from Gram-positive organisms) and locally produced cytokines (IL-8, IL-1β, TNF-α) are proposed to reach the circulation across the weakened blood–milk barrier [32,34,44,45].

**Step 2: hepatic and leukocyte sensing.** Circulating ligands and cytokines are sensed by TLR4 (and TLR2) on monocytes, Kupffer cells, and hepatocytes, activating NF-κB and the release of TNF-α, IL-1β, and IL-6 [32,66]; IL-6 drives the hepatic acute-phase response (haptoglobin, serum amyloid A, LBP) [71,72]. In the present data, TNF-α, IL-6, serum amyloid A, and haptoglobin rose progressively from −8 wk toward diagnosis (Section 4.1).

**Step 3: metabolic reprogramming.** Cytokine and TLR signaling shifts activated immune cells toward HIF-1α-dependent aerobic glycolysis (lactate, succinate) [65,66], induces IDO1 (raising the Kyn/Trp ratio) [67,68,69], activates phospholipase A_2_ and sphingomyelin turnover (lysophosphatidylcholine and sphingomyelin changes) [70], and mobilizes muscle amino acids (branched-chain amino acids, lysine). TNF-α and ceramides also impair insulin signaling, and cytokine-induced sickness behavior lowers feed intake. These are the changes detected in serum, urine, and milk from −8 wk onward (Section 3.3, Section 3.4, Section 4.1 and Section 5.1).

**Step 4: calving and expansion of exposure.** At calving, the cortisol surge, the abrupt rise in nutrient demand for milk synthesis, and reduced intake coincide with new microbial exposures: continuing mammary infection, ruminal and hindgut LPS under concentrate-rich rations [49,50,51,52,53,54], and uterine LPS and lipoteichoic acid from the contaminated postpartum uterus [62,63,64]. Innate immune function, including neutrophil function, is impaired during this period [82].

**Step 5: organ-specific clinical exits.** Which disease appears is proposed to depend on the organ system that fails first under the shared load. In the liver, inflammation-associated insulin resistance, reduced intake, and hepatic lipid accumulation favor excessive ketogenesis and clinical ketosis [43,79]. For calcium homeostasis, intramammary LPS challenge lowers circulating calcium [59], and peripartum hypocalcemia can be interpreted as part of the host response to LPS [36], which may progress to milk fever when intestinal and skeletal calcium supply cannot keep pace with colostrum synthesis. In the claw, inflammatory and vasoactive mediators are proposed to disturb dermal perfusion and the integrity of the suspensory apparatus, predisposing to laminitis-related lameness [32,35]. In the uterus, impaired neutrophil function delays detachment of the fetal membranes (retained placenta) [82] and permits bacterial overgrowth and uterine LPS accumulation (metritis) [62,63,64]. In the mammary gland, impaired local defense and tolerance-like reprogramming of mammary leukocytes favor persistence of low-grade infection (subclinical mastitis) [73,88]. Because the upstream load is shared, one cow may pass through more than one of these exits, which is the proposed basis of comorbidity.

**Step 6: persistence after clinical recovery.** Tolerance-like reprogramming of innate immune cells [73,88] and continued uterine and ruminal exposure may sustain a metabolic deviation after clinical recovery, as observed at +8 wk (Section 4.4).

Each step yields a measurable prediction: LPS and lipoteichoic acid in mammary secretion and mammary venous blood at dry-off (step 1); a prepartum rise in cytokines and acute-phase proteins that precedes the metabolic changes in the same cows (steps 2 and 3); and a reduction in the combined incidence of all six diseases after interventions directed at step 1 (step 5).

### 6.7. Alternative and Complementary Explanations

The present data do not exclude other explanations that could generate similar prepartum signatures. Three deserve specific consideration, together with the possibility that they interact with, rather than replace, the proposed LPS mechanism.

**Glucocorticoid-driven metabolic reprogramming.** Plasma cortisol rises in late gestation and peaks at calving, and prepartum cortisol, haptoglobin, and non-esterified fatty acid concentrations have been associated with postpartum health status [91]; abrupt dry-off has also been followed by increased fecal glucocorticoid metabolites, particularly in higher-yielding cows [92]. Glucocorticoids stimulate muscle proteolysis [93], which could raise circulating branched-chain amino acids and lysine; they induce hepatic tryptophan 2,3-dioxygenase, which raises the Kyn/Trp ratio independently of IDO1 [94]; and they influence hepatic one-carbon and sphingolipid metabolism. Several members of the conserved signature (Table 2) could therefore reflect endocrine rather than inflammatory input. Three observations argue against a purely endocrine explanation, although none is decisive. First, cortisol rises most sharply at calving, whereas cross-disease coherence was already high at −8 wk and peaked at −4 wk. Second, the prepartum signature included pro-inflammatory cytokines and acute-phase proteins, which glucocorticoids generally suppress rather than induce. Third, controls were at the same physiological stage and would share the endocrine changes of late gestation, so only a disease-associated excess of glucocorticoid exposure could produce the case–control contrasts. Because cortisol was not measured in the source studies, the extent to which endogenous glucocorticoids account for the observed signature cannot be determined here. Paired measurements of cortisol (or fecal glucocorticoid metabolites) and Kyn/Trp, together with hepatic tryptophan 2,3-dioxygenase and leukocyte IDO1 expression, would separate the two pathways.

**Pre-existing differences in nutrition, intake, or body condition.** Cows that later develop disease may already differ from healthy cows before dry-off. Lower prepartum dry-matter intake and feeding time identify cows at risk of metritis [95], and body condition at dry-off and calving affects lipomobilization, disease risk, and productivity [96]. In the present cohort, intake was lower before calving in cows that later developed retained placenta, milk fever, and subclinical mastitis (Section 4.1). Because the −8 wk sample was taken at dry-off, a disease-associated signature at that time could reflect a state established before dry-off rather than a response to involution. Two features of the data argue that differences in energy balance alone are unlikely to explain the signature: non-esterified fatty acids and β-hydroxybutyrate did not change consistently across diseases before calving, and the innate-immunity controls were matched to cases by parity and body condition score [6,18,21]. However, matching was not applied in every data layer, individual intake and body condition were not used as covariates, and no sample was available from before dry-off. Sampling before dry-off (for example, at −10 wk), with body condition, intake, and milk yield as covariates, is needed to distinguish a pre-existing state from one induced by dry-off.

**A primary gut-derived signal.** The temporal pattern is also compatible with a gut origin, in which altered ruminal or hindgut microbiota and increased intestinal permeability supply LPS or other microbial products, with the mammary gland acting as a secondary amplifier. The prepartum reduction of urinary microbial methylamines (TMAO, TMA, and dimethylamine) in all six diseases (Section 4.3) shows that microbial–host co-metabolism was already altered before calving, and hindgut LPS and intestinal permeability can change under conditions other than overt ruminal acidosis [53,55,56]. The close-up ration (30% concentrate) was below the level usually associated with ruminal acidosis [49,50], but reduced intake, ration changes, and stress around the close-up transition could still affect gut barrier function. Because the systemic metabolic response to LPS is expected to be largely independent of its anatomical origin, the present data cannot separate the two sources. Discriminating experiments include simultaneous measurement of LPS or its 3-hydroxy fatty acid markers [76,77] in mammary secretion, mammary venous and jugular blood, and feces; paired sequencing of udder-secretion and fecal microbiota; and intervention trials that target the mammary gland and the gut separately and compare their effects on the systemic signature.

**Postpartum diet.** The early-lactation ration differed from the close-up ration in ways that bear on the postpartum data. Its cereal content (about 34% of dry matter as barley, plus wheat distillers grains) was roughly three times that of the close-up ration, which is consistent with the larger ruminal LPS exposure proposed after calving (Section 1.3). It also contained blood meal (about 1.8% of ration dry matter) and rumen-protected lysine, both rich sources of lysine, and blood meal is also rich in leucine. Dietary supply may therefore have added to circulating and urinary lysine and branched-chain amino acids after calving. It cannot explain the prepartum elevations, because neither ingredient was fed before calving, and it is unlikely to explain the case–control contrasts, because diseased and control cows received the same rations and diseased cows ate less. In addition, blood meal, poultry tallow, and distillers grains are processed by-products that may carry heat-stable microbial products, including endotoxin. Feed endotoxin was not measured, so dietary exposure remains an untested additional source after calving.

**Cohort-level confounding.** Because all disease groups came from one herd and period, differences in farm, diet, or dry-off protocol between the source studies cannot explain the similarity (Section 2.2). Unmeasured factors shared by cows that became ill, such as parity within the multiparous range, disease history in the previous lactation, or milk yield at dry-off, could nevertheless contribute to a common signature. These explanations are not mutually exclusive. The LPS hypothesis is retained here as the working hypothesis because it accounts for both the inflammatory and the metabolic components of the signature, but glucocorticoid exposure, nutritional status, and gut-derived signals may act as independent contributors or as modifiers of the response to LPS, and each should be measured in future studies.

### 6.8. Limitations

Several limitations should be considered. First, this is a secondary analysis of data from a single research program and, for most layers, a single herd; the similarity statistics describe internal coherence within that program and require replication in independent herds under pre-registered protocols. Second, groups are small (six cases per disease, reduced to four for milk fever after calving), and all diseases share one group of 20 controls, which limits precision, inflates magnitude estimates, and makes the disease contrasts statistically dependent. Third, LPS, lipoteichoic acid, and cortisol were not measured in the cows, endotoxin was not measured in the feed, and no sample was collected before dry-off, so neither the proposed mammary source nor the alternatives in Section 6.7 can be tested directly. Fourth, sampling intervals were four weeks apart, and the diagnosis week fell at different times relative to calving in different diseases. In addition, all cows were vaccinated with an *Escherichia coli* J-5 bacterin at dry-off (Section 2.2), which may have modified their response to Gram-negative endotoxin, so the findings may not transfer directly to unvaccinated herds. Finally, the mechanistic assignments in Table 2 are interpretive, because none of the metabolites is specific to a single pathway.

## 7. Conclusions

Six clinically distinct peripartum diseases of dairy cattle—ketosis, lameness, milk fever, metritis, retained placenta, and subclinical mastitis—share a conserved metabolic-immune signature that is detectable in serum and urine from 8 weeks before calving and also evident in milk postpartum and that does not return to control levels by 8 weeks postpartum; that is compatible with the canonical TLR4 → IDO1/HIF-1α/mTORC1/PLA_2_ downstream program; that displays a biphasic temporal architecture consistent with mammary-origin endotoxin during the dry-off period followed after parturition by concurrent mammary, ruminal, and uterine sources contributing both LPS and Gram-positive lipoteichoic acid; and that generates coherent multi-tissue findings (B12-cobalt axis, lactate-glycolysis axis, BCAA-mTORC1 axis) that single-disease analyses were not designed to identify. Quantitatively, mean cross-disease similarity reached r = 0.81 in serum at −8 wk (0.71 after removal of the shared-control dependency), thirteen serum metabolites changed in the same direction in all six diseases, and the Kyn/Trp ratio was 2.8–4.0-fold higher than in controls, with complete separation (six cows per disease versus 20 controls). A single LPS-driven syndrome with mammary involution as its proximal trigger is a parsimonious explanation of these data, but it remains a hypothesis: LPS was not measured, the evidence is correlational, and glucocorticoid-driven reprogramming, pre-existing nutritional differences, and gut-derived signals have not been excluded. Because deviation from controls at 8 weeks postpartum was similar in magnitude to that 8 weeks before calving, the metabolic disturbance may outlast the clinical episode. Comorbidity, conventionally treated as a confounder, may be the expected phenotype of the syndrome. The framework opens specific, falsifiable next experiments—direct LPS quantification, mammary microbiome characterization, gradual-dry-off intervention trials, and Kyn/Trp screening validation—that no disease-specific framework would have suggested. Beyond dairy, the framework provides a portable analytical structure for studying low-grade endotoxemia and its multi-organ clinical expression in human postpartum, peri-operative, and metaflammatory disease.

Two practical applications follow, each requiring validation before adoption. First, a short prepartum screening panel anchored on the Kyn/Trp ratio, possibly combined with lactate and selected amino acids, could identify high-risk cows at dry-off, provided that cutoff values are validated in independent herds with adequate sample sizes. Second, dry-off management—including gradual cessation of milking, internal teat sealants and selective dry-cow therapy to limit new intramammary infection, and anti-inflammatory or LPS-binding strategies—could be evaluated in controlled trials that use the combined incidence of all six diseases as the primary endpoint.

## Figures and Tables

**Table 1:** Published studies from the author’s laboratory that provided the data re-analyzed in this paper.

Disease	Study [Ref.]	Matrix	Platform (Analytes)	Timepoints	Cases vs. Controls
Multiple ^a^	Hailemariam et al., 2014, *J. Dairy Sci.* [1]	Plasma	DI-LC-MS/MS	−4, −1, +1, +4 wk	6 vs. 6
Ketosis	Zhang et al., 2016, *Res. Vet. Sci.* [79]	Serum	Innate-immunity and metabolic panel ^b^	−8, −4 wk, Dx, +4 wk	6 vs. 6
Ketosis	Zhang et al., 2017, *Metabolomics* 13:43 [2]	Serum	DI-LC-MS/MS (128)	−8, −4 wk, Dx, +4, +8 wk	6 vs. 20
Ketosis	Zhang et al., 2017, *Metabolomics* 13:97 [3]	Serum, urine	ICP-MS (18 elements)	−8, −4 wk, Dx, +4, +8 wk	6 vs. 20
Ketosis	Zhang et al., 2021, *Front. Vet. Sci.* [4]	Urine	^1^H-NMR, DI-LC-MS/MS, GC-MS	−8, −4 wk, Dx, +4, +8 wk	6 vs. 20
Lameness	Zhang et al., 2015, *Animals* [6]	Serum	Innate-immunity and metabolic panel ^b^	−8, −4 wk, Dx, +4 wk	6 vs. 6
Lameness	Dervishi et al., 2020, *J. Proteomics* [7]	Serum	GC-MS	−8, −4 wk, Dx, +4, +8 wk	6 vs. 20
Lameness	Zhang et al., 2020, *Metabolomics* [8]	Serum	DI-LC-MS/MS (128)	−8, −4 wk, Dx, +4, +8 wk	6 vs. 20
Lameness	Zhang et al., 2020, *Dairy* [9]	Urine	^1^H-NMR (90)	−8, −4 wk, Dx, +4, +8 wk	6 vs. 20
Lameness	Eckel et al., 2020, *Animal* [10]	Urine	DI-LC-MS/MS (153)	−8, −4 wk, Dx, +4, +8 wk	6 vs. 20
Lameness	Zwierzchowski et al., 2020, *J. Agric. Food Chem.* [11]	Milk	DI-LC-MS/MS (132), ^1^H-NMR (36)	+2 wk	6 vs. 20
Milk fever	Zhang et al., 2018, *Res. Vet. Sci.* [12]	Serum	Innate-immunity and metabolic panel ^b^	−8, −4 wk, Dx, +4 wk	6 vs. 6
Milk fever	Zwierzchowski et al., 2024, *J. Vet. Intern. Med.* [13]	Serum	DI-LC-MS/MS	−8, −4 wk, Dx, +4, +8 wk	6 vs. 20 ^c^
Metritis	Dervishi et al., 2016, *Res. Vet. Sci.* [14]	Serum	Innate-immunity and metabolic panel ^b^	−8, −4 wk, Dx, +4 wk	6 vs. 6
Metritis	Zhang et al., 2017, *J. Agric. Food Chem.* [15]	Serum	DI-LC-MS/MS (186)	−8, −4 wk, Dx, +4, +8 wk	6 vs. 20
Metritis	Hailemariam et al., 2018, *Can. J. Anim. Sci.* [16]	Serum	GC-MS	−8, −4 wk, Dx	6 vs. 20
Metritis	Dervishi et al., 2018, *Metabolomics* [17]	Urine	^1^H-NMR	−8, −4 wk, Dx, +4, +8 wk	6 vs. 20 ^d^
Retained placenta	Dervishi et al., 2016, *J. Anim. Sci. Biotechnol.* [18]	Serum	Innate-immunity and metabolic panel ^b^	−8, −4 wk, Dx, +4 wk	6 vs. 6
Retained placenta	Dervishi et al., 2018, *Animal* [19]	Serum	DI-LC-MS/MS	−8, −4 wk, Dx, +4, +8 wk	6 vs. 20
Retained placenta	Zhang et al., 2021, *Metabolites* [20]	Serum	GC-MS (27)	−8, −4 wk, day of RP	6 vs. 16
Subclinical mastitis	Dervishi et al., 2015, *J. Anim. Sci. Technol.* [21]	Serum	Innate-immunity and metabolic panel ^b^	−8, −4 wk, Dx, +4 wk	6 vs. 6
Subclinical mastitis	Dervishi et al., 2017, *J. Proteome Res.* [22]	Serum	GC-MS	−8, −4 wk, Dx, +4, +8 wk	6 vs. 20
Subclinical mastitis	Zwierzchowski et al., 2020, *Res. Vet. Sci.* [23]	Urine	DI-LC-MS/MS (186)	−8, −4 wk, Dx, +4, +8 wk	6 vs. 20
Subclinical mastitis	Zhang et al., 2022, *J. Agric. Food Chem.* [24]	Serum	DI-LC-MS/MS (128)	−8, −4 wk, Dx, +4, +8 wk	6 vs. 20
Subclinical mastitis ^e^	Haxhiaj et al., 2022, *Dairy* [25]	Serum	LC-MS/MS	−8, −4 wk	10 vs. 15
Subclinical mastitis ^e^	Zwierzchowski et al., 2024, *Metabolites* [26]	Urine	LC-MS, FIA-MS/MS	−8, −4 wk	10 vs. 15

Dx, week of disease diagnosis; DI-LC-MS/MS, direct-injection/liquid chromatography–tandem mass spectrometry; GC-MS, gas chromatography–mass spectrometry; ICP-MS, inductively coupled plasma–mass spectrometry; FIA, flow-injection analysis. Numbers in parentheses are the numbers of metabolites or elements quantified. ^a^ Mastitis, metritis, retained placenta, and laminitis pooled. ^b^ IL-1, IL-6, TNF-α, haptoglobin, serum amyloid A, LPS-binding protein, lactate, non-esterified fatty acids, and β-hydroxybutyrate (LBP not measured in [79]); controls matched to cases by parity, age, and body condition score. ^c^ Two cows were culled after diagnosis, leaving four at Dx, +4, and +8 wk. ^d^ Six controls at +4 and +8 wk. ^e^ Separate commercial herd (Ponoka, Alberta); cited for context and not pooled into the cross-disease analyses.

**Table 2:** The thirteen serum metabolites altered in the same direction and significant (*p* < 0.05) in all six peripartum diseases at −8 wk pre-calving (DI-LC-MS/MS), with the kynurenine/tryptophan ratio summarized in the final row. Values are log2(disease/control); SD is across the six diseases. Values are original data of the author’s laboratory, computed in the present re-analysis from per-cow serum DI-LC-MS/MS measurements of the cohort described in Section 2.2 (six cows per disease label, 20 controls), previously reported disease-by-disease in [2,8,13,15,19,24]; no values were taken from other laboratories.

Metabolite	Mean log2FC (SD)	Range Across Six Diseases	Proposed Mechanistic Interpretation
Lysine	+2.64 (0.10)	+2.50 to +2.78	Immune substrate supply
PC aa C30:2	+2.05 (0.38)	+1.64 to +2.66	PLA_2_/membrane remodeling
lysoPC a C18:0	+1.53 (0.30)	+0.97 to +1.79	PLA_2_/membrane remodeling
Leucine	+1.52 (0.13)	+1.38 to +1.69	BCAA–mTORC1
Isoleucine	+1.47 (0.07)	+1.38 to +1.57	BCAA–mTORC1
Acetylornithine	+1.46 (0.35)	+0.85 to +1.88	Polyamine pathway (arginase/M2)
SM(OH) C24:1	+1.06 (0.18)	+0.83 to +1.32	Sphingomyelin–ceramide flux
SM(OH) C22:1	+1.01 (0.17)	+0.82 to +1.31	Sphingomyelin–ceramide flux
Sarcosine	+1.00 (0.23)	+0.65 to +1.28	One-carbon (GNMT) flux
Kynurenine	+0.96 (0.15)	+0.72 to +1.11	IDO1 product
SM(OH) C22:2	+0.79 (0.15)	+0.61 to +0.99	Sphingomyelin–ceramide flux
Tryptophan	−0.91 (0.12)	−1.10 to −0.79	IDO1 substrate
Asparagine	−0.94 (0.38)	−1.62 to −0.57	Immune substrate consumption
Kyn/Trp ratio	2.8–4.0× elevated	complete separation, *p* = 8.7 × 10^−6^	Canonical IDO1 activity readout

## Data Availability

The data supporting the findings of this study are derived from the primary studies cited herein [1,2,3,4,5,6,7,8,9,10,11,12,13,14,15,16,17,18,19,20,21,22,23,24,25,26,79] and from unpublished data generated in the same cohort (Table 1). Compiled effect-size tables and analysis scripts are available from the corresponding author upon reasonable request.

## Supplementary Materials

The following supporting information can be downloaded at: https://www.mdpi.com/article/10.3390/metabo16100743/s1, Table S1: Composition of the close-up (prepartum) and early-lactation rations fed to the cows of the source studies.
