# Supplementary material for: Peripartum Disease in Dairy Cows as a Single LPS-Driven Metabolic-Immune Syndrome: Convergent Metabolomic Evidence Across Six Clinical Presentations
Source: Metabolites. 2026 Oct 2;16(10):743. doi: 10.3390/metabo16100743 (PMC13707186; doi:10.3390/metabo16100743)
Supplement: Supplementary file 1 [file metabolites-16-00743-s001.zip › metabolites-4549307-supplementary.pdf]

Supplementary Materials

Peripartum Disease in Dairy Cows as a Single LPS-Driven Metabolic-Immune Syndrome: Convergent Metabolomic Evidence Across Six Clinical Presentations

Table S1. Composition of the close-up (prepartum) and early-lactation rations fed to the cows of the source studies (reproduced from Zhang et al. [6]).

(a) Close-up diet (CUD)

**Ingredient**	**% of DM**
Alfalfa hay	10.0
Barley silage	60.0
CUD grain mix	30.0
**CUD grain mix composition**	**% of mix**
Barley grain, rolled	39.5815
Soybean hulls, ground	33.0
Canola meal	17.0
Limestone	3.7
Scale molasses (60:40)	2.5
Magnesium chloride	1.645
Mag Ox-56% ^4	0.54
Salt	0.54
Flo-bond mycotoxin binder	0.5
Custom TM Complex Premix ^2	0.33
Ruminant TM Pak ^1	0.2775
Selenium 1000 mg/kg (UNscr FineCr)	0.2
Vitamin E 50% Ads ^5	0.18
Vitamin A/D3-1000-200 ^3	0.006

(b) Early-lactation diet

**Ingredient**	**% of DM**
Alfalfa hay	9.59
Barley silage	30.24
Alfalfa silage	9.64
High 16% dairy ration	50.5
**Dairy ration composition**	**% of mix**
Barley grain, rolled	39.91
Barley grain, ground	27.50
Wheat distillers grain (50:50)	10.00
Amino Plus (high-bypass soy) [7]	8.00
Blood meal	3.50
Energizer RP10	2.75
Limestone	1.70
Scale molasses (60:40)	1.25
Soybean meal, 47.5%	1.25
Di-calcium phosphate 21%	1.00
Sodium bicarbonate	0.80
Salt	0.51
Poultry tallow	0.50
Mag Ox-56% [6]	0.43
AminoShure-L [4]	0.33

Diamond V XPC [5]	0.13
Ruminant TM Pak [2]	0.11
Nutri A-Z C Dry	0.10
Selenium 1000 mg/kg (UNscr FineCr)	0.07
Custom TM Complex premix [3]	0.07
ADE Vit Pak-30 Natural E [1]	0.05
Vit D 10,000 KIU/kg	0.02
Dairy Xtract	0.02
Vitamin E 50% Ads [8]	0.01
Biotin 2% (Rovimix H-2) [9]	0.01

Footnotes, (a): [1] Premix containing cobalt, copper, iodine, manganese, and zinc. [2] Custom product supplying organic sources of cobalt, copper, manganese, and zinc. [3] Vitamin A acetate (retinyl acetate) and vitamin D3 (cholecalciferol). [4] 56% magnesium guarantee. [5] Contains 226,800 IU of vitamin E per pound.

Footnotes, (b): [1] Premix containing vitamins A, D3, and E. [2] Premix containing cobalt, copper, iodine, manganese, and zinc. [3] Custom product supplying organic sources of cobalt, copper, manganese, and zinc. [4] Hydrogenated vegetable oil and L-lysine monohydrochloride (Halchemix, Port Perry, ON, Canada). [5] Concentrated yeast (Diamond V Mills, Cedar Rapids, IA, USA). [6] 56% magnesium guarantee. [7] High by-pass soybean meal. [8] Contains 226,800 IU of vitamin E per pound. [9] DSM Nutritional Products (Parsippany, NJ, USA). DM, dry matter; CUD, close-up diet.
